# A general synthesis approach for amorphous noble metal nanosheets

**DOI:** 10.1038/s41467-019-12859-2

**Published:** 2019-10-24

**Authors:** Geng Wu, Xusheng Zheng, Peixin Cui, Hongyu Jiang, Xiaoqian Wang, Yunteng Qu, Wenxing Chen, Yue Lin, Hai Li, Xiao Han, Yanmin Hu, Peigen Liu, Qinghua Zhang, Jingjie Ge, Yancai Yao, Rongbo Sun, Yuen Wu, Lin Gu, Xun Hong, Yadong Li

**Affiliations:** 1 Hefei National Laboratory for Physical Sciences at the Microscale, Department of Applied Chemistry, Center of Advanced Nanocatalysis (CAN), University of Science and Technology of China, 230026 Hefei, Anhui People’s Republic of China; 2 National Synchrotron Radiation Laboratory (NSRL), University of Science and Technology of China, 230029 Hefei, Anhui People’s Republic of China; 3 Key Laboratory of Soil Environment and Pollution Remediation, Institute of Soil Science, Chinese Academy of Sciences, 210008 Nanjing, People’s Republic of China; 4 Institute of Physics, Chinese Academy of Sciences, 100190 Beijing, People’s Republic of China; 5 Beijing Key Laboratory of Construction Tailorable Advanced Functional Materials and Green Applications, School of Materials Science and Engineering, Beijing Institute of Technology, 100081 Beijing, People’s Republic of China; 6 Hefei National Laboratory for Physical Sciences at the Microscale, University of Science and Technology of China, 230026 Hefei, People’s Republic of China; 7 Key Laboratory of Flexible Electronics (KLOFE) and Institute of Advanced Materials (IAM), Jiangsu National Synergetic In-novation Center for Advanced Materials (SICAM), Nanjing Technology University, 211816 Nanjing, Jiangsu People’s Republic of China; 8 Department of Chemistry, Tsinghua University, 100084 Beijing, People’s Republic of China

**Keywords:** Catalyst synthesis, Electrocatalysis, Two-dimensional materials

## Abstract

Noble metal nanomaterials have been widely used as catalysts. Common techniques for the synthesis of noble metal often result in crystalline nanostructures. The synthesis of amorphous noble metal nanostructures remains a substantial challenge. We present a general route for preparing dozens of different amorphous noble metal nanosheets with thickness less than 10 nm by directly annealing the mixture of metal acetylacetonate and alkali salts. Tuning atom arrangement of the noble metals enables to optimize their catalytic properties. Amorphous Ir nanosheets exhibit a superior performance for oxygen evolution reaction under acidic media, achieving 2.5-fold, 17.6-fold improvement in mass activity (at 1.53 V vs. reversible hydrogen electrode) over crystalline Ir nanosheets and commercial IrO_2_ catalyst, respectively. In situ X-ray absorption fine structure spectra indicate the valance state of Ir increased to less than + 4 during the oxygen evolution reaction process and recover to its initial state after the reaction.

## Abstract (web summary)

While noble metal usage in catalysis is ubiquitous, the metals’ scarcity necessitates new materials designs for efficient utilization. Here, authors report a general strategy to prepare amorphous noble metal nanosheets and find the nanomaterials to act as efficient water-splitting electrocatalysts.

## Introduction

Noble metal nanomaterials possess potential applications in catalysis^1–3^, energy storage, and conversion^4–6^. For example, Ir and its oxide nanomaterials are unanimously considered as the most promising catalysts for oxygen evolution reaction (OER) under acidic media, which is the primary bottleneck in electrolysis of water due to the intrinsically sluggish kinetics^7–9^. Unfortunately, the scarcity and expensiveness of noble metals greatly hamper their widespread application. Therefore, tremendous efforts have been devoted to constructing more active noble metal catalysts by increasing the number of active sites or optimizing the intrinsic activity^7^. To this end, how the size^10^, shape^11,12^, and crystal phase^13–15^ of noble nanostructures influence their catalytic performance have been well investigated. As the adsorption/desorption kinetics of intermediate species depend well on the surface structure of catalysts^16^, the construction of catalysts surface structure^17–19^, especially on the atomic level, has emerged as an efficient and successful strategy to further enhance the catalytic performance of noble metal nanomaterials.

Distinctive from crystalline materials with translational periodicity, the special disorder atomic structure with the lack of long-range rotational and translational symmetry^20,21^ endows amorphous materials with unique properties^22–24^. For example, amorphous materials possess superior elastic strain performance, which is attributed to the chemical fluctuations and local topological in the amorphous structure^22^. Moreover, the large amount of randomly oriented bonds enable amorphous materials with abundant defects and coordination-unsaturated sites on the surface^25–28^, potentially providing superior catalytic performance than crystalline counterparts. For instance, amorphization can efficiently promote the intrinsic properties of Pd_3_P_2_S_8_ for the electrocatalytic hydrogen evolution reaction and amorphous gelled FeCoW oxyhydroxides exhibit a remarkable performance for OER in alkaline electrolyte compared with crystalline counterparts^29,30^. Unfortunately, since the strong and isotropic nature of metallic bonds^31^, noble metal nanomaterials synthesized by conventional strategies are usually crystalline^32^. Therefore, the synthesis of amorphous noble metal nanomaterials where elemental composition, material size, and shape can be precisely controlled remains substantial challenges.

**Fig. 1:**
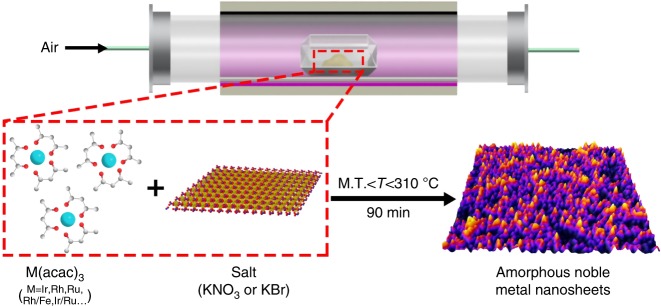
Schematic illustration of the general synthetic process for amorphous noble metal NSs. Note: M.T. is the melting point of metal acetylacetonate

Herein, we demonstrate a general and facile method to synthesize amorphous noble metal nanosheets (NSs) via directly annealing metal acetylacetonates with alkali salt (Fig. 1 and Supplementary Table 1). The synthetic temperature is situated between the melt point of metal acetylacetonate and that of alkali salt. After removing alkali salt with water, high-yield amorphous noble metal-based NSs have been obtained, including but not limited to monometal NSs (Ir NSs, Rh NSs, Ru NSs), bimetal NSs (RhFe NSs, IrRu NSs), and trimetal NSs (IrRhRu NSs). The obtained amorphous Ir NSs achieve exceptional catalytic activity when benchmarked again commercial catalysts for electrochemical OER under acidic media.

## Results

### Synthesis and characterization of amorphous Ir NSs

**Fig. 2:**
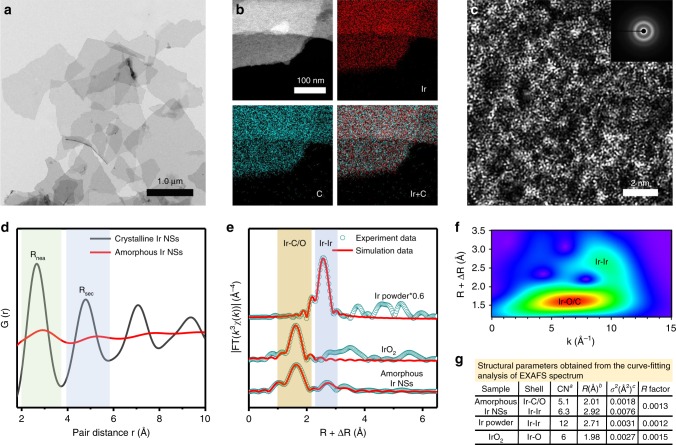
Characterizations of amorphous Ir NSs. **a** TEM, **b** HAADF-STEM image and the corresponding EDS elemental mapping, **c** aberration-corrected HAADF-STEM image of amorphous Ir NSs. The inset in **c** shows the SAED pattern. **d** Radial distribution functions of amorphous Ir NSs and crystalline Ir NSs. **e** Fourier transforms of k_3_-weighted Ir L_3_-edge EXAFS experimental data for amorphous Ir NSs, Ir powder and IrO_2_. **f** Wavelet transform of Ir L_3_-edge EXAFS data for amorphous Ir NSs. **g** Structural parameters obtained from the curve-fitting analysis of the EXAFS spectrum. Note: ^a^CN is the coordination number. ^b^*R* is interatomic distance (the bond length between central atoms and surrounding coordination atoms). ^c^*σ*^2^ is Debye–Waller factor (a measure of thermal and static disorder in absorber–scatterer distances)

Amorphous Ir NSs have been prepared by annealing the mixture of iridium acetylacetonates (Ir(acac)_3_) and alkali nitrate (KNO_3_) in air. Typical transmission electron microscopy (TEM) image (Fig. 2a) and scanning electron microscopy (SEM) image (Supplementary Fig. 1) reveal the lateral size of obtained NSs controls up to a few micrometers. Atomic force microscopy (AFM) image (Supplementary Fig. 2) displays that the thickness of NSs is ~7.2 nm. High-angle annular dark-field scanning TEM (HAADF-STEM) image and the corresponding energy-dispersive X-ray spectroscopy (STEM-EDS) elemental mapping (Fig. 2b) confirm that Ir and C are homogeneously distributed within whole NSs. Quantitative EDS spectrum (Supplementary Fig. 3) reveals that the Ir content in NSs is up to 84 wt%. Aberration-corrected HAADF-STEM image (Fig. 2c) shows the disordered atomic structure, verifying the amorphous feature, which accords with the diffractive halo-like selected area electron diffraction (SAED) pattern (the inset in Fig. 2c). These data coincide well with the X-ray diffraction (XRD) pattern (Supplementary Fig. 4), in which diffraction peaks for neither Ir nor IrO_2_ crystal can be detected. As depicted in X-ray photoelectron spectroscopy (XPS) spectra (Supplementary Fig. 5), the peaks located at 61.0 and 63.9 eV are assigned to Ir (4*f*_7/2_) and Ir (4*f*_5/2_) of Ir^0^ and the peaks located at 62.1 and 65.0 eV are assigned to Ir (4*f*_7/2_) and Ir (4*f*_5/2_) of Ir^4+^, which can be contributed to partial oxidation of Ir under air atmosphere, respectively^33,34^. To quantify the local structural characterization of samples, the radial distribution function (RDF) was obtained from SAED patterns of amorphous and crystalline Ir NSs by using the PASAD tools^35^. As shown in the RDF plots (Fig. 2d), the first two peaks (*R*_nea_ and *R*_sec_) reflect to the average distance of the nearest and the second nearest-neighbor Ir atoms, respectively. Notably, in the case of amorphous NSs, the *R*_nea_ peak position is shifted to higher distances (2.92 Å) compared to the crystalline NSs (2.68 Å). Furthermore, distinct from those for the crystalline NSs, the *R*_nea_ and *R*_sec_ peaks in amorphous NSs are broadened and shifted toward higher distances. The observed deviation of RDF peak positions and their significant broadening for amorphous NSs indicate that the corresponding atomic structure has a poor periodicity. Extended X-ray absorption fine structure (EXAFS) spectrum and X-ray absorption near-edge structure (XANES) spectrum of Ir L_3_-edge were employed to further investigate the atomic structure of amorphous NSs (Fig. 2e–g, Supplementary Figs. 6, 7). The peak around 2.92 Å attributed to Ir-Ir bonds in amorphous NSs is slightly larger than that in Ir powder (2.71 Å), presumably resulting from the loose packing characteristic of non-crystalline state^25,36^. On the basis of the curve-fitting analysis of the EXAFS spectrum (Fig. 2e, g), the coordination number of Ir-C/O and Ir-Ir in the amorphous Ir NSs are 5.1 and 6.3, respectively. Moreover, the wavelet spectrum of amorphous Ir NSs (Fig. 2f) contains two obvious intensity of Ir-C/O and Ir-Ir coordination, in line with the corresponding curve-fitting results.

### Characterization of other amorphous noble metal NSs

**Fig. 3:**
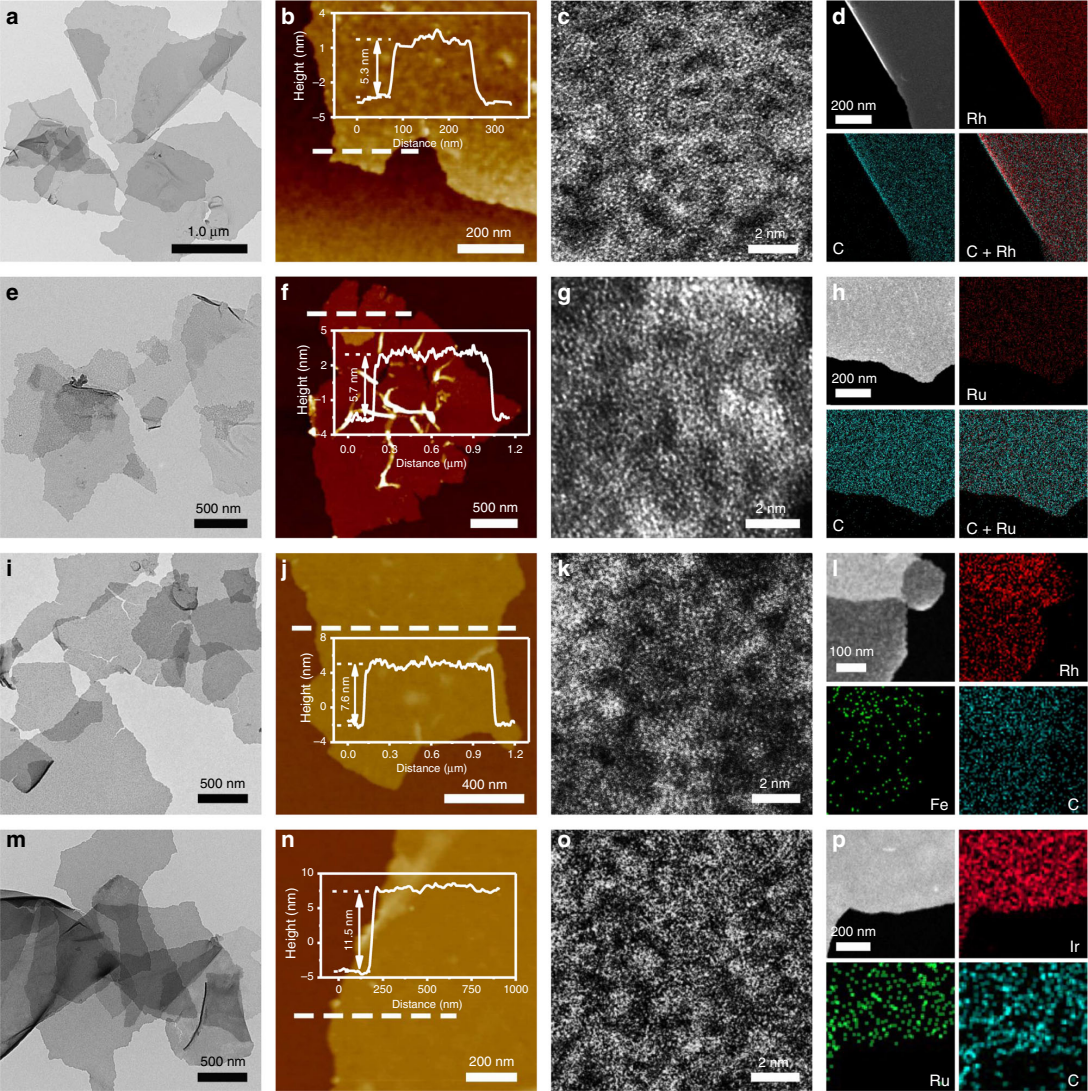
Characterizations of other amorphous noble metal NSs. **a**, **e**, **i**, **m** TEM, **b**, **f**, **j**, **n** AFM, **c**, **g**, **k**, **o** aberration-corrected HAADF-STEM image, and **d**, **h**, **l**, **p** HAADF-STEM image and the corresponding EDS elemental mapping of amorphous Rh NSs, Ru NSs, RhFe NSs, and IrRu NSs

To further expand applicability of the synthetic method, amorphous Rh NSs with a thickness of about 5.3 nm and amorphous Ru NSs with a thickness of about 5.7 nm were effectively prepared and corroborated by TEM, SEM, and HAADF-STEM images (Fig. 3a–c, e–g) as well as XRD pattern (Supplementary Fig. 8). Similarly, the homogeneous spatial distributions of metal element and carbon in amorphous noble metal NSs can be also confirmed by EDS elemental mappings (Fig. 3d, h and Supplementary Fig. 9, 10). Besides monometal amorphous NSs, bimetal amorphous NSs can be also fabricated by the similar approach. For example, amorphous RhFe bimetallic NSs with a thickness of about 7.6 nm and IrRu bimetallic NSs with a thickness of about 11.5 nm were successfully obtained (Fig. 3i–p and Supplementary Figs. 8, 11, 12). Analogously, a wide variety of bimetal and even trimetal amorphous NSs, such as amorphous IrFe (IrNi, IrCo) NSs, RhNi (RhCo, RhRu) NSs, RuFe (RuNi, RuCo) NSs, IrRh NSs, and IrRhRu NSs, can also be conveniently prepared (Supplementary Figs. 13–15). Furthermore, the thickness control of amorphous NSs could be also achieved, as demonstrated by the synthesis of amorphous Ir NSs with a thickness of 3.6 nm (Supplementary Figs. 16–18).

Taking amorphous Ir NSs as a representative example, control experiment with different parameters, including temperature, salt substrate, atmosphere, and annealing time, were further carried out for an in-depth understanding of the growth processes. First, temperature is critical for the formation of amorphous NSs, which must be situated between the melt point of metal acetylacetonate and that of alkali salt. The annealing temperature above the melt point of alkali salt leads to the production of crystalline materials (Supplementary Fig. 19), while an annealing temperature below the melt point of metal acetylacetonate cannot drive the reaction. Second, alkali salt is quite essential for controlling the formation of amorphous NSs. In the absence of alkali salt, crystalline Ir nanoparticles were obtained via directly annealing Ir(acac)_3_ (Supplementary Fig. 20), indicating that noble metal can be in situ reduced by carbon originated from the thermal decomposition of metal acetylacetonate. When changing alkali salt substrate from KNO_3_ to NaCl (or KBr), crystalline Ir NSs were synthesized instead of amorphous products (Supplementary Figs. 21, 22). The change of structure might be attributed to the different diffusion rate of Ir atoms on the substrate surface, while the low diffusion rate is beneficial to form amorphous structure during the nucleation process^37,38^. Third, carbon-supported crystalline Ir nanoparticles were prepared by changing the atmosphere from air to argon (Supplementary Fig. 23). Combined by EXAFS spectra and XPS data, the Ir-C/O coordination that existed in amorphous Ir NSs might help in maintaining the amorphous structure. Furthermore, crystalline Ir NSs were formed through extending heat time (Supplementary Fig. 24).

### Electrocatalytic activity of amorphous Ir NSs towards OER

**Fig. 4:**
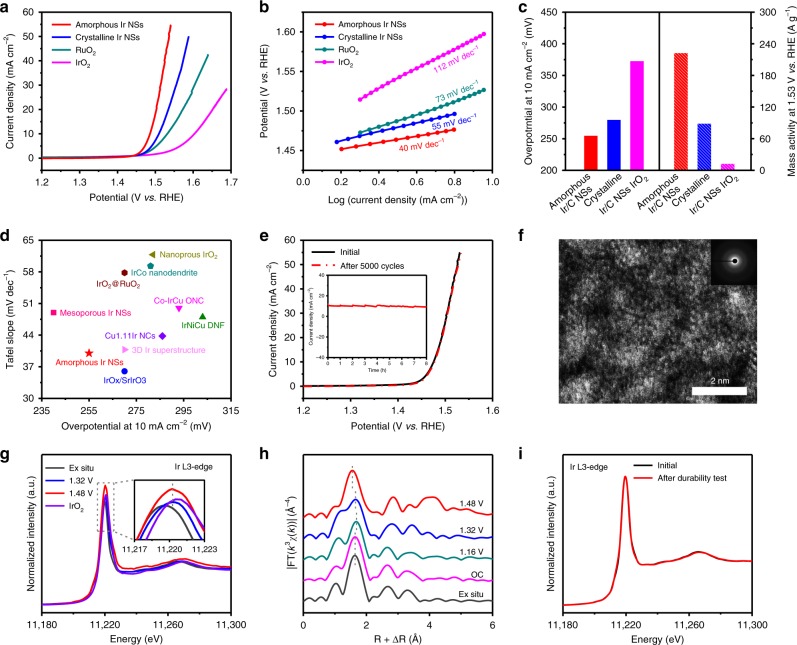
Electrochemical OER performance and operando X-ray absorption spectroscopy of amorphous Ir NSs. **a** Polarization curves of amorphous Ir NSs, crystalline Ir NSs, commercial RuO_2_ and IrO_2_ catalysts, respectively. **b** Corresponding Tafel plots of amorphous Ir NSs, crystalline Ir NSs, commercial RuO_2_ and IrO_2_ catalysts, respectively. **c** Overpotentials at 10 mA cm^−2^ (left axis) and mass activity at 1.53 V (vs. RHE) (right axis) of amorphous Ir NSs, crystalline Ir NSs, and commercial IrO_2_ catalyst, respectively. **d** Comparison with different representative catalysts under acidic media. **e** Polarization curves of amorphous Ir NSs before and after 5000 cycles. The inset in **e** shows chronoamperometry measurement of the amorphous Ir NSs at an overpotential of 255 mV for a 8-h test. Note: All the measurements were performed in O_2_-saturated 0.1 M HClO_4_ solution. **f** Atomic resolution HAADF-STEM image of the amorphous Ir NSs after durability test. The inset in **f** shows the corresponding SAED pattern. **g**, **h** In situ XAFS spectra change of the Ir L_3_-edge. **i** XANES spectrum of amorphous Ir NSs after durability test

As a proof of concept, we chose acidic OER to evaluate the electrocatalytic performance of amorphous Ir NSs. Crystalline Ir NSs (see Supplementary Methods for experimental details and Supplementary Fig. 22), and commercial RuO_2_ and IrO_2_ catalysts (Supplementary Fig. 25) are employed as references. As shown in polarization curves normalized by glassy carbon electrode (GCE) geometric area (0.196 cm^2^) (Fig. 4a), the amorphous Ir NSs require an overpotential of only 255 mV to achieve a current density of 10 mA cm^−2^, which is lower than that of crystalline Ir NSs (280 mV), RuO_2_ (301 mV), and IrO_2_ (373 mV) catalysts. To evaluate the kinetic behaviors of the electrocatalysts, the Tafel plots of elerctrocatalysts (Fig. 4b) exhibit slopes of 40, 55, 73, and 112 mV dec^−1^ for amorphous Ir NSs, crystalline Ir NSs, RuO_2_, and IrO_2_ catalysts, respectively. The considerably smaller slope achieved by the amorphous Ir NSs indicates significantly improved kinetics towards electrochemical OER. Remarkably, amorphous Ir NSs deliver a high mass activity of 221.8 A g^−1^ at an overpotential of 300 mV (Fig. 4c and Supplementary Fig. 26), which is 2.5 times and 17.6 times larger than that of the crystalline Ir NSs (88.7 A g^−1^) and the IrO_2_ (12.6 A g^−1^), respectively. Moreover, the amorphous Ir NSs exhibit almost the lowest overpotential for a current density of 10 mA cm^−2^ and the smallest Tafel slope compared with previously reported noble metal electrocatalysts (Fig. 4d and Supplementary Table 2). In order to illustrate the superior OER activity of amorphous Ir NSs, electrochemically active surface area (ECSA) and electrochemical impedance spectroscopy (EIS) were tested. The ECSA can be obtained by calculating double-layer capacitance (*C*_dl_)^39,40^. As shown in Supplementary Figs. 27, 28, the amorphous Ir NSs present a rather high ECSA, revealing that amorphous Ir NSs have abundant OER active sites. Furthermore, the Nyquist plots (Supplementary Fig. 29) show that the amorphous Ir NSs have the lowest charge-transfer resistance among all the tested catalysts, verifying the faster charge transfer process for amorphous Ir NSs. Furthermore, the turnover frequency (TOF) of catalysts were also calculated at different applied potentials^41^ (Supplementary Fig. 30). Amorphous Ir NSs show a TOF value of 0.16 s^−1^ at an overpotential of 300 mV, which is 3.1, 9.9, and 22.8 times larger than that of the crystalline Ir NSs (0.052 s^−1^), RuO_2_ (0.016 s^−1^), and IrO_2_ (0.007 s^−1^), respectively. Besides activity, stability is another important parameter to evaluate the performance of OER electrocatalysts. Surprisingly, the OER activity of amorphous Ir NSs display negligible degradation even after 5000 cycles (Fig. 4e), indicating the excellent stability of amorphous Ir NSs. This observation coincides well with chronoamperometry measurement (inset of Fig. 4e), in which the current density of amorphous Ir NSs at an overpotential of 255 mV sustained ~90% after a 8-h test. Furthermore, XRD pattern and aberration-corrected HAADF-STEM analysis after long-term durability test further exhibit ignorable change in the amorphous atomic structure (Fig. 4f and Supplementary Fig. 31).

### In situ XAFS studies of amorphous Ir NSs during OER process

To track the evolution process of the atomic structures of Ir during OER process, in situ XAFS spectra of amorphous Ir NSs were carried out under different applied potentials^42,43^. As depicted in the in situ XANES spectra (Supplementary Figs. 33, 34), the location of white line peak is shifted to the higher energy from ex situ condition to 1.32 V (vs. reversible hydrogen electrode (RHE)), indicating an increase for the valance state of Ir. Moreover, the location of white line peak remains almost unchanged with the potential increased from 1.32 to 1.48 V (vs. RHE), while the current density raised from 0.5 mA to 7.6 mA cm^−2^ (Fig. 4g), which implies an invariability in the oxidation state of Ir during the OER process. Notably, the valance state of Ir has always been maintained lower than +4 under all tested potentials. Furthermore, the local environment evolutions of Ir can be investigated by in situ EXAFS spectrum (Fig. 4h). The distance of the first coordination peak (Ir-C/O) slightly increased from the open-circuit (OC) condition to 1.16 V (vs. RHE), probably owing to the adsorption of water molecule from the electrolyte. The bond length of Ir-C/O decreased while the applied potential increased from 1.16 to 1.48 V (vs. RHE), which is possibly attributed to the formation of intermediate species (such as OOH*) or oxygen molecule on the surface^44^. Importantly, the valance state of Ir and the distance of Ir-C/O for amorphous Ir NSs can recover to its initial state after restoring back to the original potential (Supplementary Fig. 35). Moreover, the XAFS spectra of Ir L_3_-edge remains unchanged after a 8-h durability test (Fig. 4i and Supplementary Fig. 36), further revealing the stability of amorphous Ir NSs.

## Discussion

In summary, we demonstrate a general and efficient method to fabricate amorphous noble metal NSs by directly annealing the mixture of metal acetylacetonate and alkali salts. Besides monometal NSs, dozens of different amorphous bimetal NSs and trimetal NSs can be successfully and conveniently prepared. Impressively, the as-prepared amorphous Ir NSs were used as electrocatalyst for OER under acidic media, exhibiting superior electrocatalytic performance compared to crystalline Ir NSs and most reported noble metal electrocatalysts. In situ XAFS spectra reveal that the valance state of Ir has always been maintained lower than +4 during the OER catalysis process. Furthermore, the amorphous atomic structure remains stable after durability test, as demonstrated by aberration-corrected HAADF-STEM image combined with XAFS studies. These findings show that amorphous noble metal materials can not only boost the catalytic activity because of abundant active sites and unique atomic structure but also endow superior catalytic capability that conventional corresponding crystalline catalysts do not afford.

## Methods

### Chemicals

Iridium(III) 2,4-pentanedionate (Ir(acac)_3_), rhodium(III) 2,4-pentanedionate (Rh(acac)_3_), iron(III) 2,4-pentanedionate (Fe(acac)_3_), nickel(II) 2,4-pentanedionate (Ni(acac)_2_), cobalt(III) 2,4-pentanedionate (Co(acac)_3_) were all purchased from Alfa Aesar. Ruthenium acetylacetonate (Ru(acac)_3_) were purchased from Adamas-Beta. Sodium nitrate (NaNO_3_), potassium nitrate (KNO_3_), sodium chloride (NaCl), potassium bromide (KBr), and alcohol were all obtained from Sinopharm Chemical Reagent Co., Ltd (Shanghai, China). Ruthenium oxide (RuO_2_) and iridium oxide (IrO_2_) were purchased from Alfa Aesar. Deionized (DI) water from Milli-Q System (Millipore, Billerica, MA) was used in all our experiments. Nafion solution (5 wt%) was purchased from Aldrich. All chemicals were used as received without further purification.

### Synthesis of amorphous Ir NSs

In a typical synthesis of amorphous Ir NSs, 10 mg Ir(acac)_3_ and 20 mg KNO_3_ were dissolved into 7 mL mixture solution (*V*_ethanol_/*V*_DI water_ = 6/1) under magnetic stirring. The mixture solution was dried to obtain the mixed powder. The mixture powder was heated to 300 °C for 90 min at the heating rate of 5 °C min^−1^ under air in a tube furnace and then naturally cooled to room temperature, yielding the representative samples. The as-obtained products were washed several times with the DI water–ethanol mixing solution.

### Synthesis of amorphous Rh NSs, Ru NSs, and RhRu NSs

In a typical synthesis of amorphous Rh NSs, 8 mg Rh(acac)_3_ and 15 mg KBr were dissolved into 7 mL mixture solution (*V*/*V*_DI_ water = 6/1) under magnetic stirring. The mixture solution was dried to obtain the mixed powder. The mixture powder was heated to 280–290 °C for 90 min under air in a tube furnace and then naturally cooled to room temperature. The as-obtained products were washed several times with the DI water–ethanol mixing solution. The amorphous Ru NSs were obtained using the same process as that of amorphous Rh NSs, except that 8 mg Rh(acac)_3_ was replaced with 8 mg Ru(acac)_3_. The amorphous RhRu NSs were obtained using the same process as that of amorphous Rh NSs, except the addition of 0.4 mg Ru(acac)_3_.

### Synthesis of amorphous IrRu NSs, IrRh NSs, and IrRhRu NSs

In a typical process of amorphous IrRu NSs, 10 mg Ir(acac)_3_, 0.4 mg Ru(acac)_3_, and 20 mg KNO_3_ were dissolved into 7 mL mixture solution (*V*_ethanol_/*V*_DI water_ = 6/1) under magnetic stirring. The mixture solution was dried to obtain the mixed powder. The mixture powder was heated to 280–290 °C for 90 min under air in a tube furnace and then naturally cooled to room temperature. The as-obtained products were washed several times with DI water–ethanol mixture. The amorphous IrRh NSs were obtained using the same process as that of amorphous IrRu NSs, except that 0.4 mg Ru(acac)_3_ was replaced with 0.4 mg Rh(acac)_3_. The amorphous IrRhRu NSs were obtained using the same process as that of amorphous IrRu NSs, except the addition of 0.4 mg Rh(acac)_3_.

### Synthesis of amorphous RhFe NSs, RhNi NSs, RhCo NSs, RuFe NSs, RuNi NSs RuCo NSs, IrNi NSs, IrFe NSs, and IrCo NSs

In a typical synthesis of amorphous RhFe NSs, 8 mg Rh(acac)_3_, 0.35 mg Fe(acac)_3_, and 15 mg KBr were dissolved into 7 mL mixture solution (*V*_ethanol_/*V*_DI water_ = 6/1) under magnetic stirring. The mixture solution was dried to obtain the mixed powder. The mixture powder was heated to 270–280 °C for 90 min under air in a tube furnace and then naturally cooled to room temperature. The as-obtained products were washed several times with DI water–ethanol mixture. The amorphous RhNi NSs (RhCo NSs) were obtained using the same process as that of amorphous RhFe NSs, except that 0.35 mg Fe(acac)_3_ was replaced with 0.25 mg Ni(acac)_2_ (0.35 mg Co(acac)_3_). The amorphous RuFe NSs (RuNi NSs, RhCo NSs) were obtained using the same process as that of amorphous RhFe NSs (RhNi NSs, RhCo NSs), except that 8 mg Rh(acac)_3_ was replaced with 8 mg Ru(acac)_3_. The amorphous IrFe NSs (IrNi NSs, IrCo NSs) were obtained using the same process as that of amorphous RhFe NSs (RhNi NSs, RhCo NSs), except that 8 mg Rh(acac)_3_ was replaced with 10 mg Ir(acac)_3_.

### Synthesis of amorphous Ir NSs with a thickness of 3.6 nm and crystalline Ir NSs

The amorphous Ir NSs with a thickness of 3.6 nm were obtained using the same process as that of amorphous Ir NSs, except that 20 mg KNO_3_ was replaced with 18 mg NaNO_3_. In addition, the crystalline Ir NSs were obtained using the same process as that of amorphous Ir NSs, except that 20 mg KNO_3_ was replaced with 15 mg NaCl (KBr).

### Characterization

TEM images of all samples were recorded on Hitachi H-7700 operated at 100 kV. The SEM and the EDS of samples were carried out by Genimi SEM 500. Aberration-corrected HAADF-STEM images of samples were recorded on JEOL JEM-2010 LaB_6_ high-resolution TEM and double Cs-corrected JEOL JEM-ARM200CF STEM, which were operated at 200 kV. The XRD patterns of samples were conducted on Rigaku Miniflex-600 operating at the voltage of 40 kV and the current of 15 mA with Cu Kα radiation (*λ* = 1.5406 Å). XPS was measured at beamline BL10B of National Synchrotron Radiation Laboratory (NSRL) of China using Mg Kα (*hν* = 1248.6 eV) radiation source. AFM image was captured by Dimension ICON with Nano Scope V controller (Bruker) in Scan Asyst and tapping mode. The X-ray absorption find structure data (Ir L_3_-edge) was collected at BL14W1 beamline of Shanghai Synchrotron Radiation Facility (SSRF) operated at 3.5 GeV under “top-up” mode with a constant current of 240 mA. The acquired EXAFS data were processed according to the standard procedures using the ATHENA module implemented in the IFEFFIT software packages.

### Electrochemical measurements for OER

All the electrochemical experiments were conducted on the CHI 760E electrochemical workstation (Shanghai Chenhua, China) in a conventional three-electrode system at room temperature. Samples were tested on the GCE as the working electrode (WE), Ag/AgCl as the reference electrodes, and Pt wire as the counter electrode (CE). The catalyst solutions were prepared by mixing 2.0 mg catalysts with 2.0 mg carbon black (Cabot Vulcan XC-72) in a solution containing 490 μL of ethanol, 490 μL of H_2_O, and 20 μL of 5 wt% Nafion solution by sonication to form homogeneous catalyst inks. Then, 20 μL well-dispersed catalyst ink was carefully dropped onto the polished glassy carbon rotating disk electrode with drying naturally for testing.

OER tests were conducted in O_2_-saturated 0.1 M HClO_4_ solution with a scan rate of 5 mV s^−1^ at 1600 r.p.m. on a rotating electrode. All potentials were referenced to an RHE with IR correction, where the R was referred to the ohmic resistance arising from the electrolyte/contact resistance of the setup and measured by EIS. EIS measurements were carried out from 100 kHz to 0.1 Hz on a rotation electrode under 1600 r.p.m.

### ECSA calculation

The ECSA was estimated from the electrochemical double-layer capacitance (*C*_dl_) of the catalytic surface. The *C*_dl_ was determined by measuring the non-Faradaic capacitive current charging from the scan rates dependence of cyclic voltammograms. The double layer capacitance (*C*_dl_) is obtained from the charge current (*i*_c_) as function of the scan rate (*ν*), which is equal to the slope based on Eq. (1) shown as follows:

1 $$C_{\mathrm{dl}} = \frac{{i_{\mathrm{c}}}}{v}.$$

ECSA is calculated using Eq. (2), whereas the specific capacitance (*C*_s_) is 0.035 mF cm^−2^ in 0.1 M HClO_4_ aqueous solution:

2 $${\mathrm{ECSA}} = \frac{{C_{\mathrm{dl}}}}{{C_{\mathrm{S}} \times m_{\mathrm{catalyst}}}}.$$

### TOF calculation

The TOF was calculated based on the method reported in previous works. This calculation assumes 100% Faradaic efficiency:

3 $${\mathrm{TOF}} = \frac{{N_{\mathrm{O}_2}}}{{N_{\mathrm{metal}}}},$$

where $N_{\mathrm{O}_2}$ is the number of O_2_ turnovers, calculated using the following formula:

4 $$N_{\mathrm{O}_2} = \frac{{j (\frac{A}{{\mathrm{cm}}^2}) \times {\mathrm{Scm}}_{\mathrm{oxide}}^2 \times 1\frac{\mathrm{C}}{\mathrm{s}} \times 1\,{\mathrm{mol}}\,{\mathrm{e}}^ - }}{{96,485\;{\mathrm{C}} \times 4e^ - }} \times N_{\mathrm{A}},$$

where *j* is the measured current density, *A* is the surface area of electrode, and *N*_A_ is Avogadro constant (6.02 × 10^23^ mol^−1^).

The number of metal sites (*N*_metal_) is only calculated the metal (Ir or Ru) number sites in tested catalyst.

### In situ XAFS measurements

Electrochemical measurements were conducted on a computer-controlled electrochemical analyzer (Supplementary Fig. 32). Catalyst-modified carbon paper was used as the WE, Pt wire as the counter electrode (CE) and Ag/AgCl electrode as the reference electrode (RE). In situ XAFS was used to probe the valence state and coordination environment for amorphous Ir NSs during OER process. The dilute catalyst slurry (50 μl ink) was evently distributed on the carbon paper (3 cm × 3 cm).

### Collection and analysis of RDF

The microstructure and SAED of the samples were investigated by double Cs-corrected JEOL JEM-ARM200CF scanning TEM that was operated at 200 kV. The conversion of SAED pattern (Fig. 2c and Supplementary Fig. 22) into the radial distribution function g(r) was conducted using the freely available PASAD-tools package for the GATAN Digital micrograph software.

## Supplementary information

Supplementary Info

## Data Availability

The data that support the findings of this study are available from the corresponding authors upon reasonable request.
