# Supplementary material for: A general synthesis approach for amorphous noble metal nanosheets
Source: Nat Commun. 2019 Oct 24;10:4855. doi: 10.1038/s41467-019-12859-2 (PMC6813339; doi:10.1038/s41467-019-12859-2)
Supplement: Supplementary file 1 — Supplementary Info [file 41467_2019_12859_MOESM1_ESM.pdf]

Supplementary Information

A general synthesis approach for amorphous noble metal nanosheets

Wu et al.

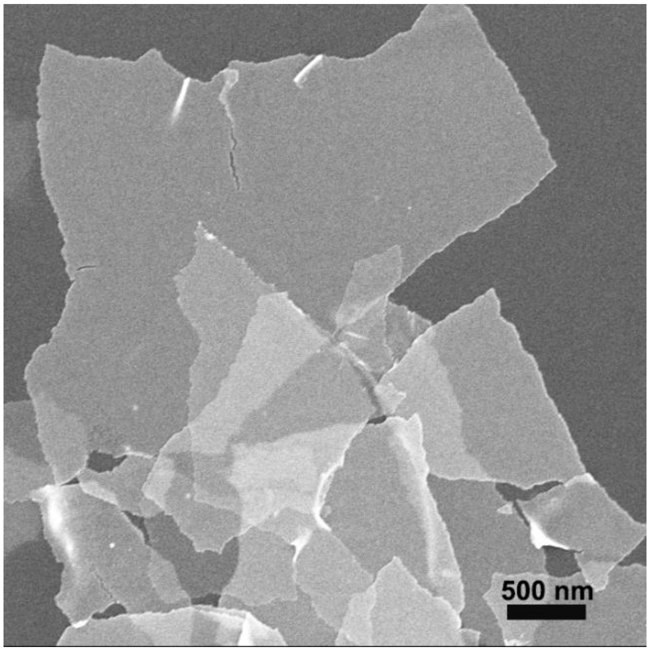

Supplementary Figure 1. SEM image of amorphous Ir NSs.

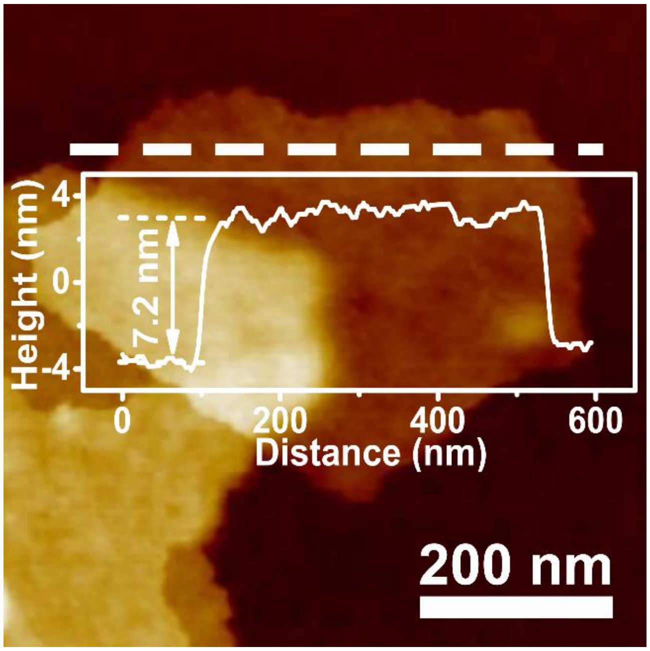

Supplementary Figure 2. AFM image of amorphous Ir NSs.

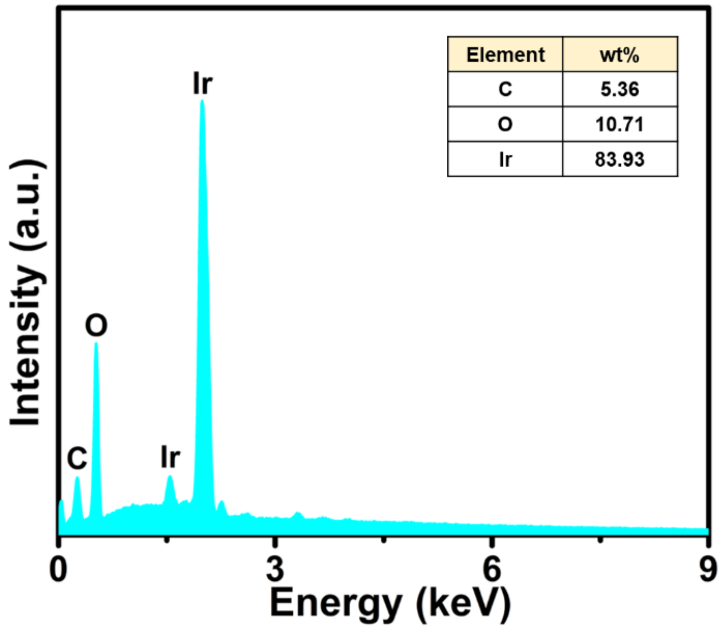

Supplementary Figure 3. EDS spectrum of amorphous Ir NSs.

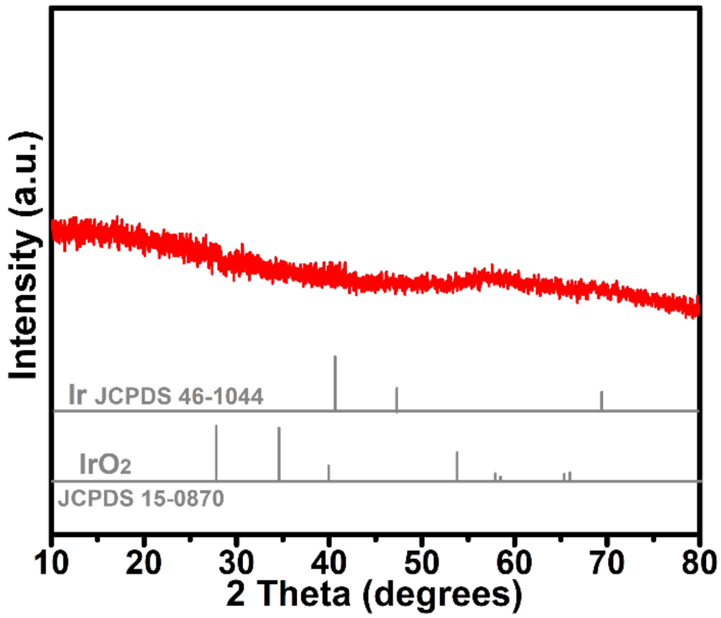

Supplementary Figure 4. XRD pattern of amorphous Ir NSs.

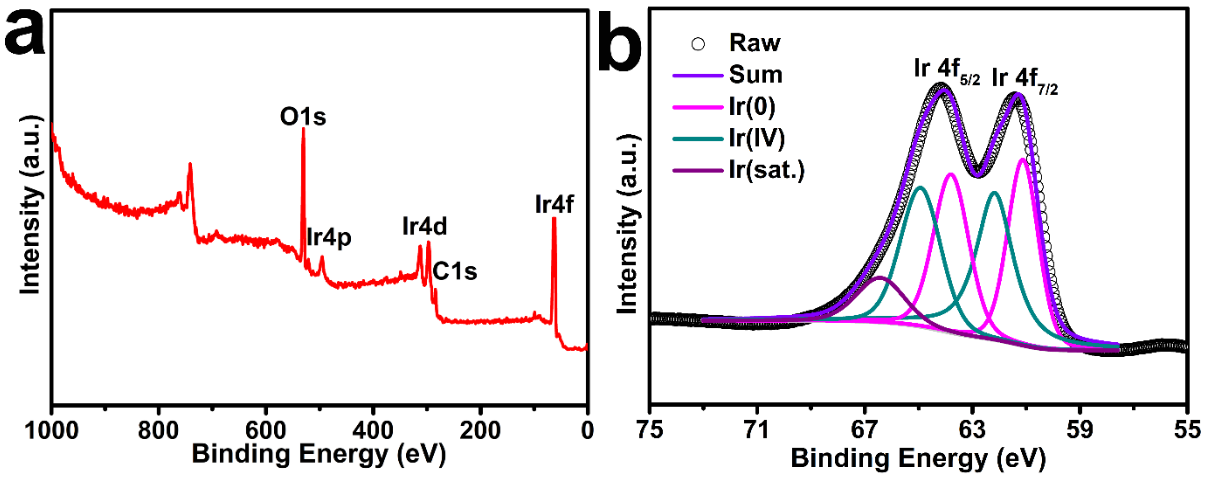

Supplementary Figure 5. XPS spectra of amorphous Ir NSs. (a) XPS survey spectrum and (b) high-resolution Ir4f XPS spectrum of amorphous Ir NSs.

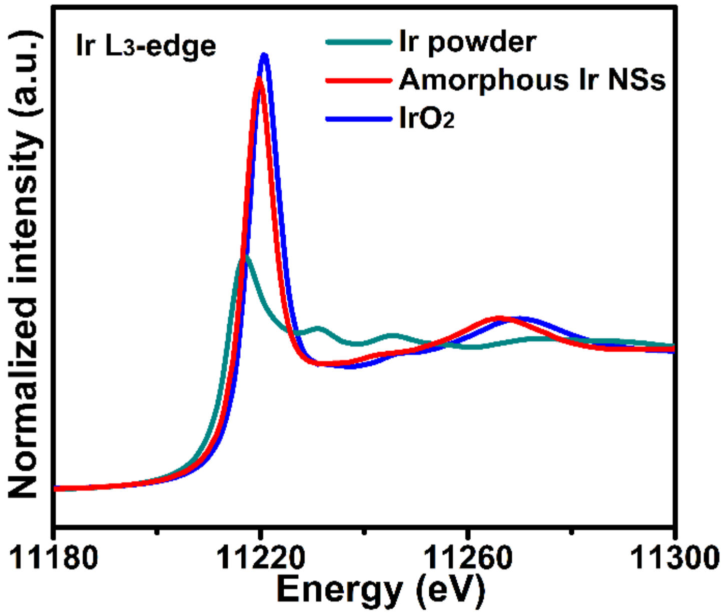

Supplementary Figure 6. XANES spectrum of amorphous Ir NSs. The location of white line peak for amorphous Ir NSs is situated between that of Ir powder and IrO_2, indicating that the valance state of Ir for amorphous Ir NSs is lower than +4, in line with the XPS data.

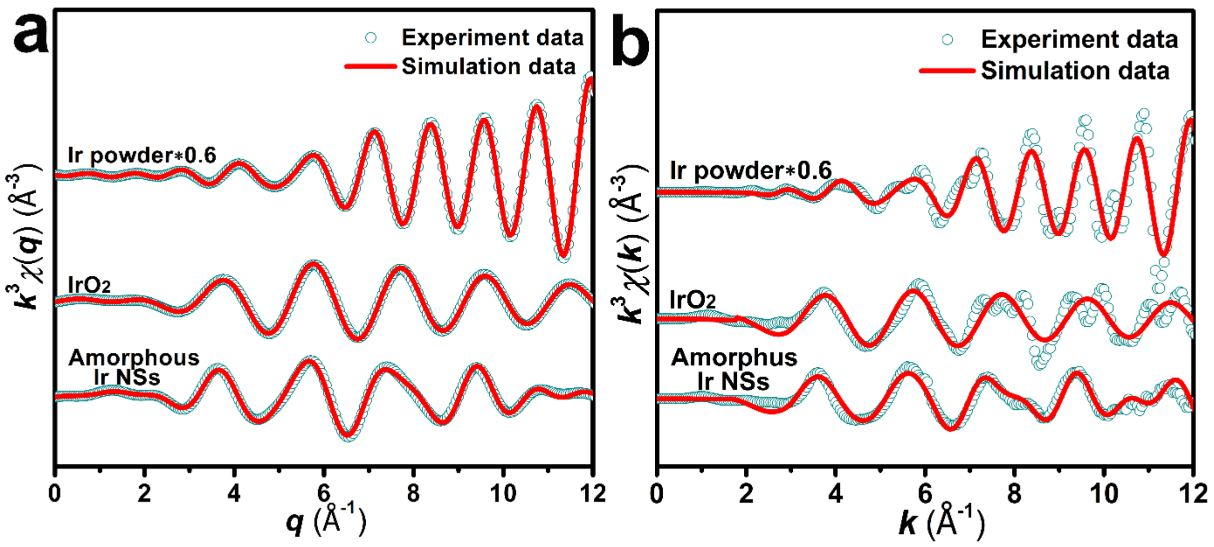

Supplementary Figure 7. Inverse FT-EXAFS Fitting result and EXAFS oscillations of amorphous Ir NSs. (a) Inverse FT-EXAFS Fitting result and (b) Ir L_3-edge EXAFS oscillations of Ir powder, amorphous Ir NSs and IrO_2.

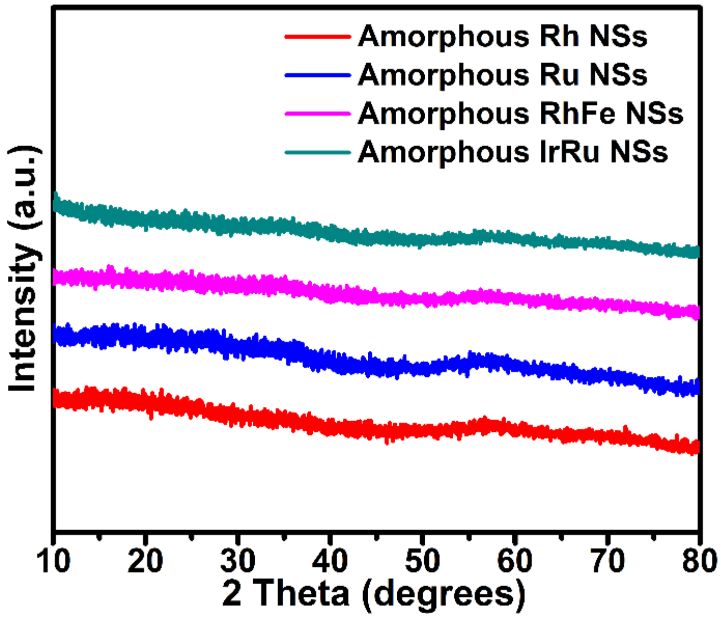

Supplementary Figure 8. XRD pattern of amorphous Rh NSs, Ru NSs, RhFe NSs, IrRu NSs.

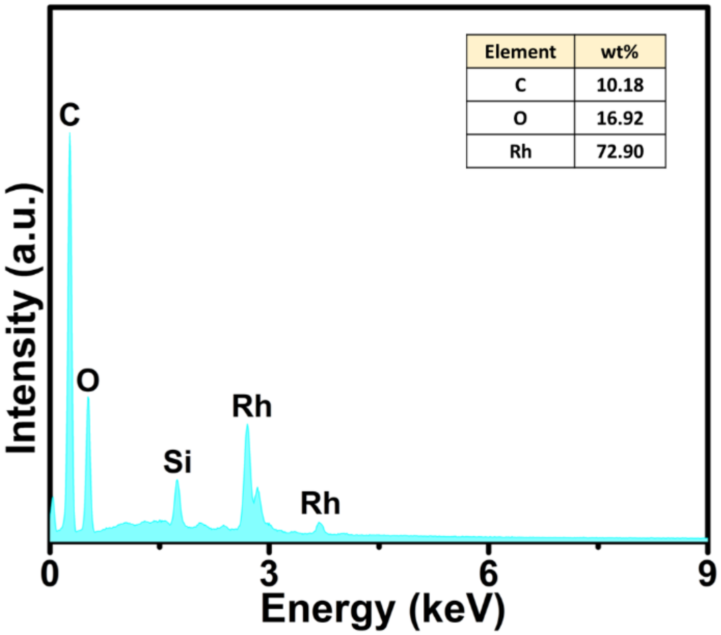

Supplementary Figure 9. EDS spectrum of amorphous Rh NSs.

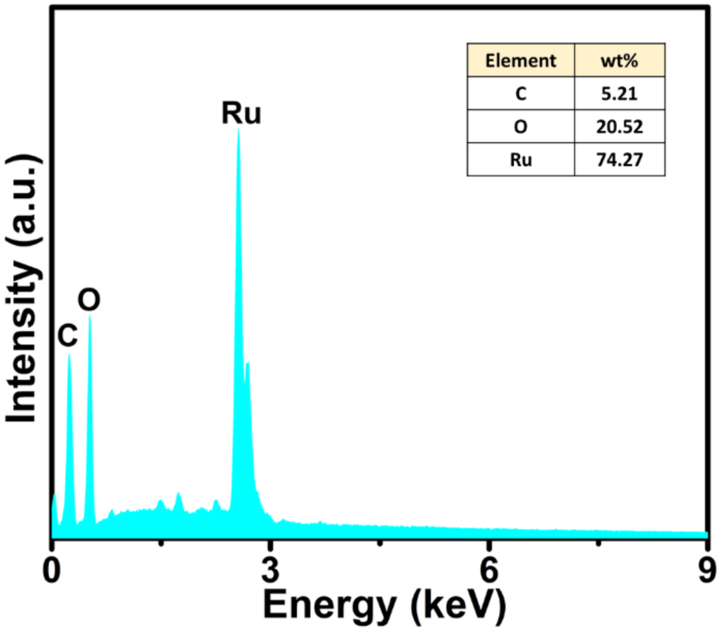

Supplementary Figure 10. EDS spectrum of amorphous Ru NSs.

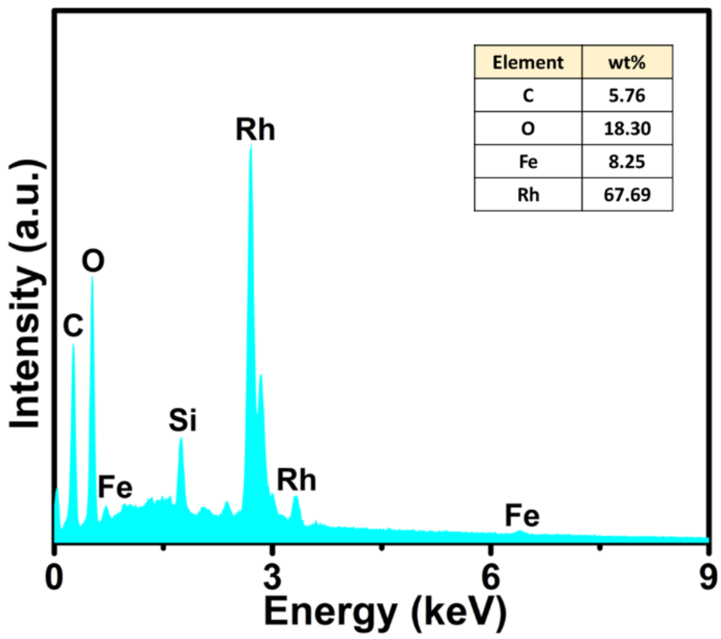

Supplementary Figure 11. EDS spectrum of amorphous RhFe NSs.

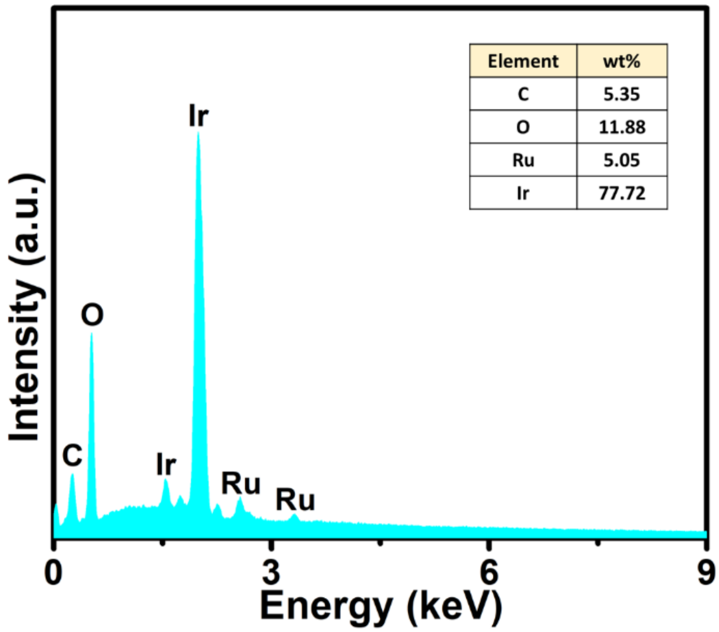

Supplementary Figure 12. EDS spectrum of amorphous IrRu NSs.

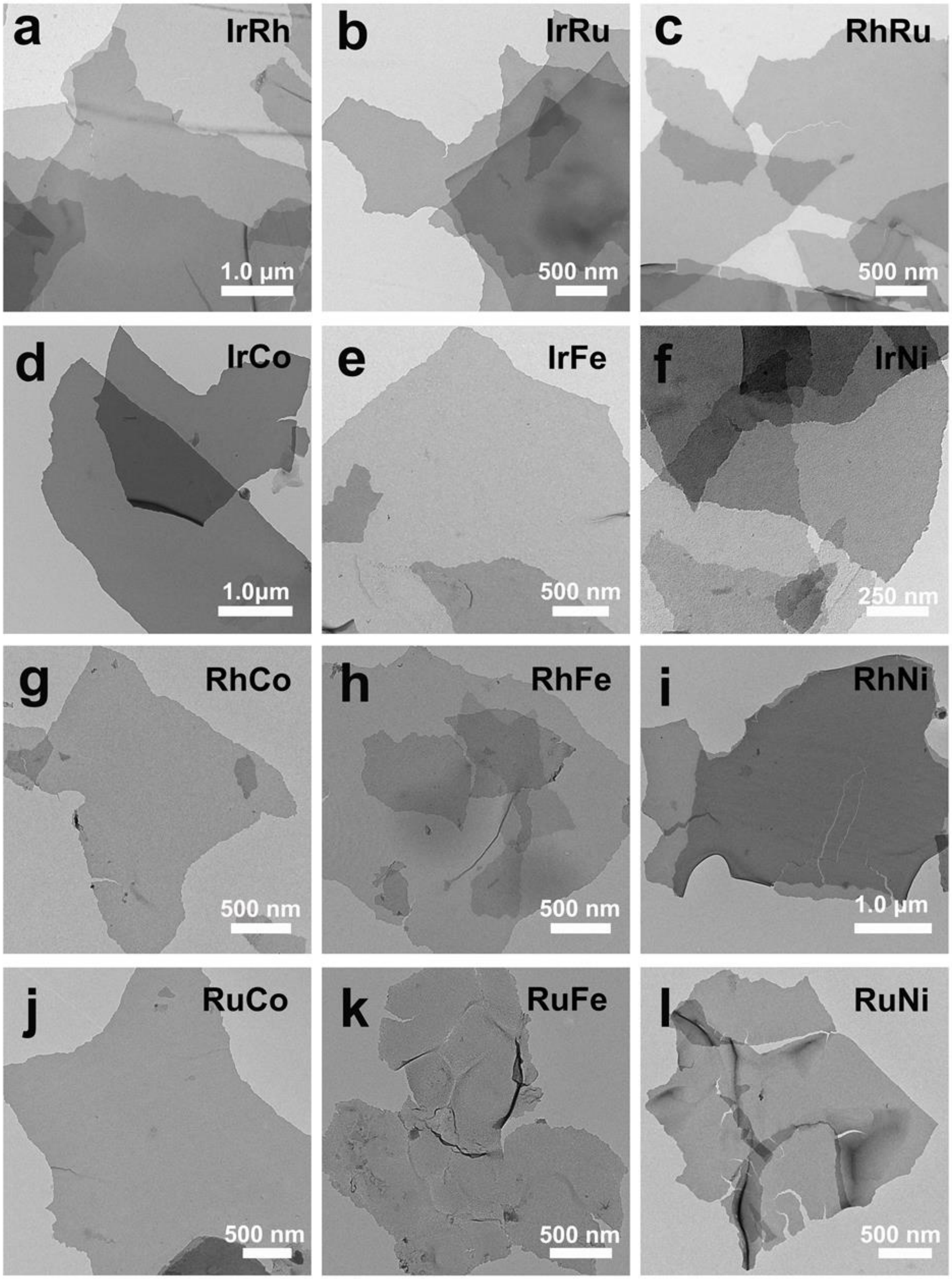

Supplementary Figure 13. TEM images of amorphous bimetal NSs.

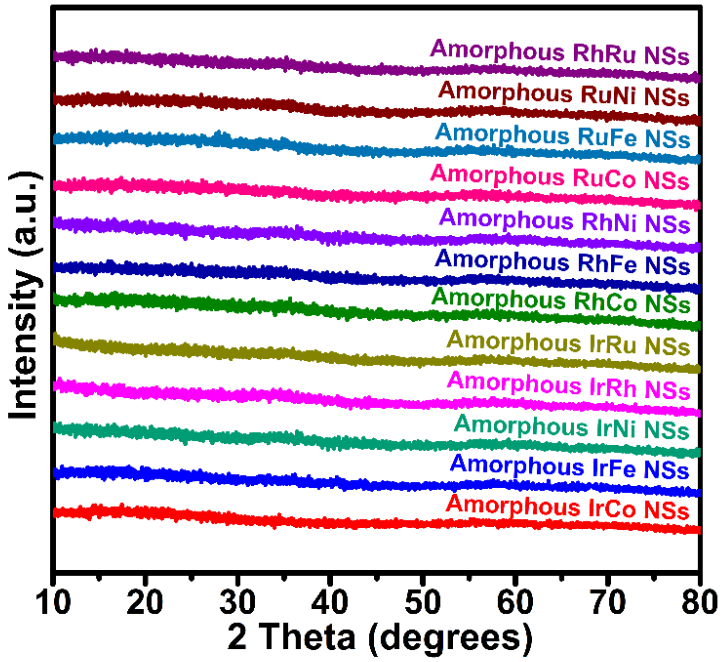

Supplementary Figure 14. XRD pattern of amorphous bimetal NS.

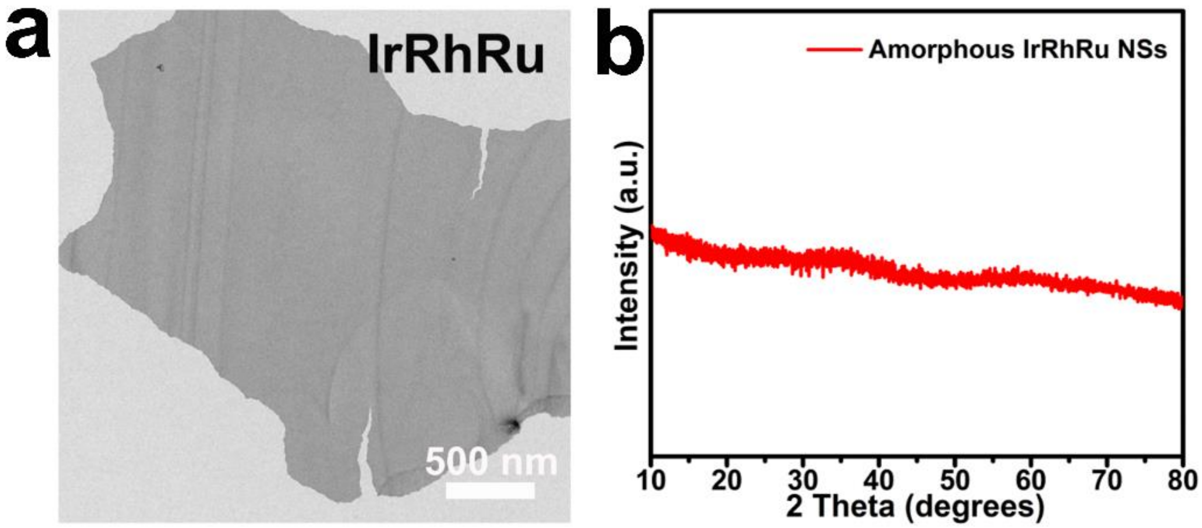

Supplementary Figure 15. Characterizations of amorphous IrRhRu NSs. (a)TEM image and (b) XRD pattern of amorphous IrRhRu NSs.

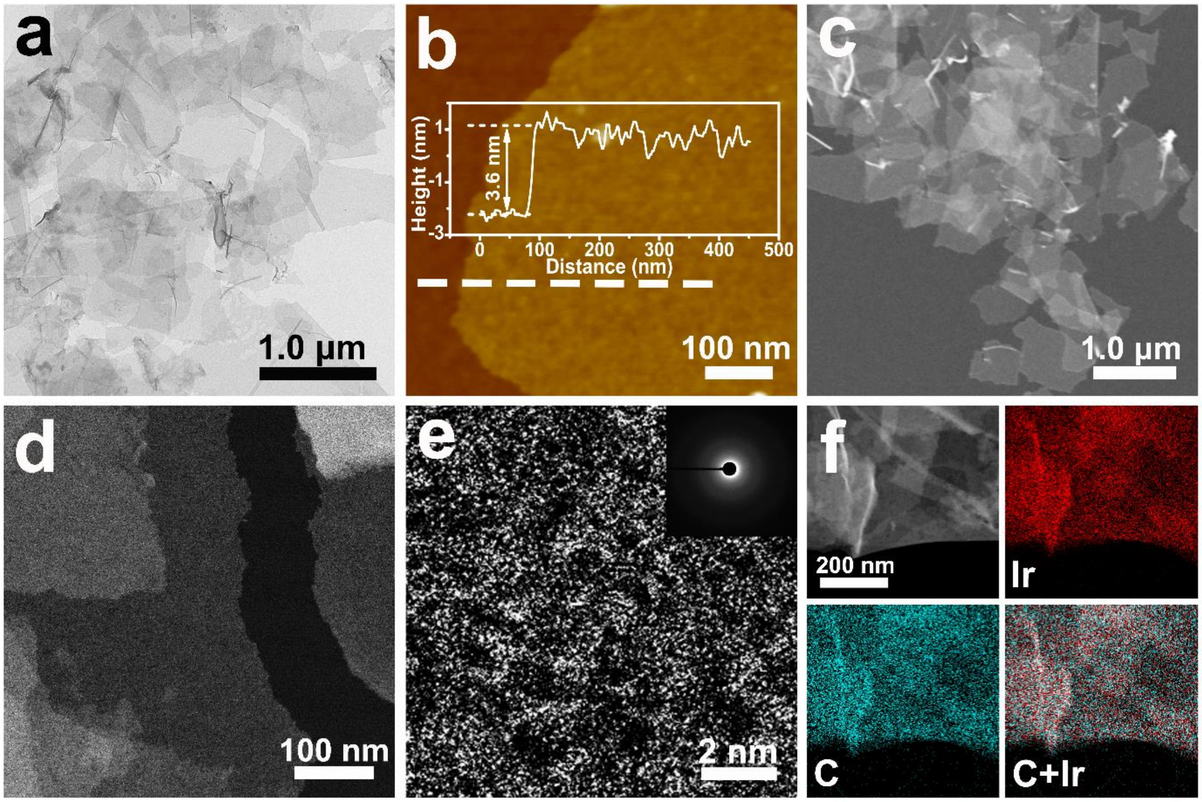

Supplementary Figure 16. Characterizations of amorphous Ir NSs with a thickness of 3.6 nm. (a)TEM, (b)AFM, (c)SEM, (d) High resolution TEM, (e) Aberration-corrected HAADF-STEM image, (f) HAADF-STEM image and the corresponding EDS elemental mapping of amorphous Ir NSs with a thickness of 3.6 nm. The inset in (e) shows the SAED pattern.

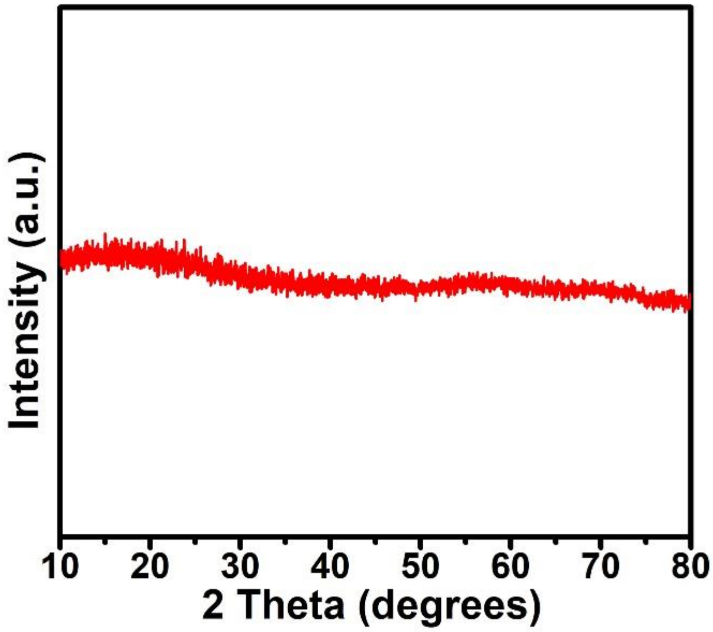

Supplementary Figure 17. XRD pattern of amorphous Ir NSs with a thickness of 3.6 nm.

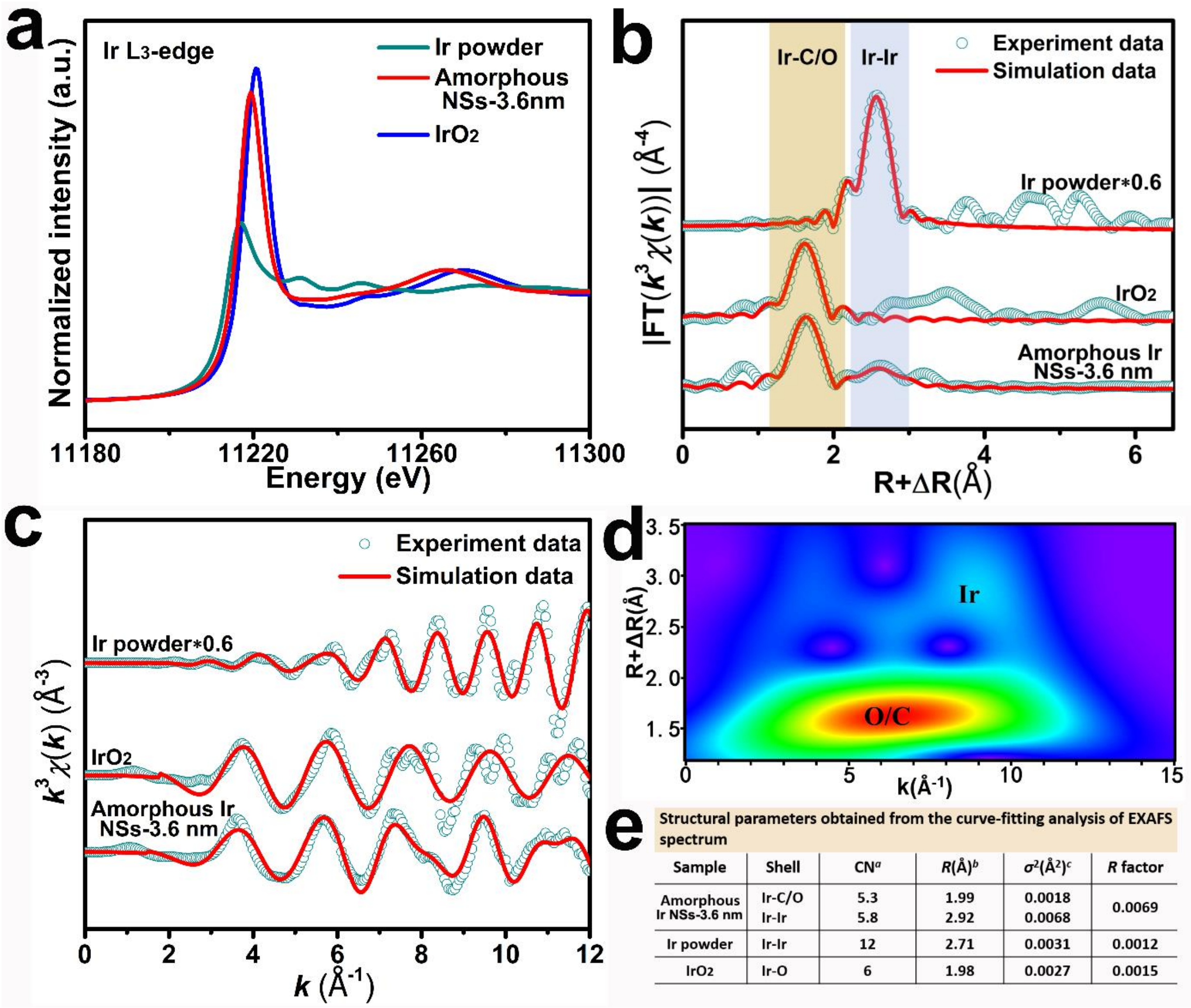

e Structural parameters obtained from the curve-fitting analysis of EXAFS spectrum

Sample	Shell	CN[a]	R(Å)[b]	σ^2(Å^2)[c]	R factor
Amorphous Ir NSs-3.6 nm	Ir-C/O<br>Ir-Ir	5.3<br>5.8	1.99<br>2.92	0.0018<br>0.0068	0.0069
Ir powder	Ir-Ir	12	2.71	0.0031	0.0012
IrO_2	Ir-O	6	1.98	0.0027	0.0015

Supplementary Figure 18. XAFS spectra of amorphous Ir NSs with a thickness of 3.6 nm. (a) XANES spectrum, (b) Fourier transforms of k^3-weighted Ir L_3-edge EXAFS experimental data, (c) Ir L_3-edge EXAFS oscillations and (d) Wavelet transform of Ir L_3-edge EXAFS data for amorphous Ir NSs with a thickness of 3.6 nm. (e) Structural parameters obtained from the curve-fitting analysis of the EXAFS spectrum.

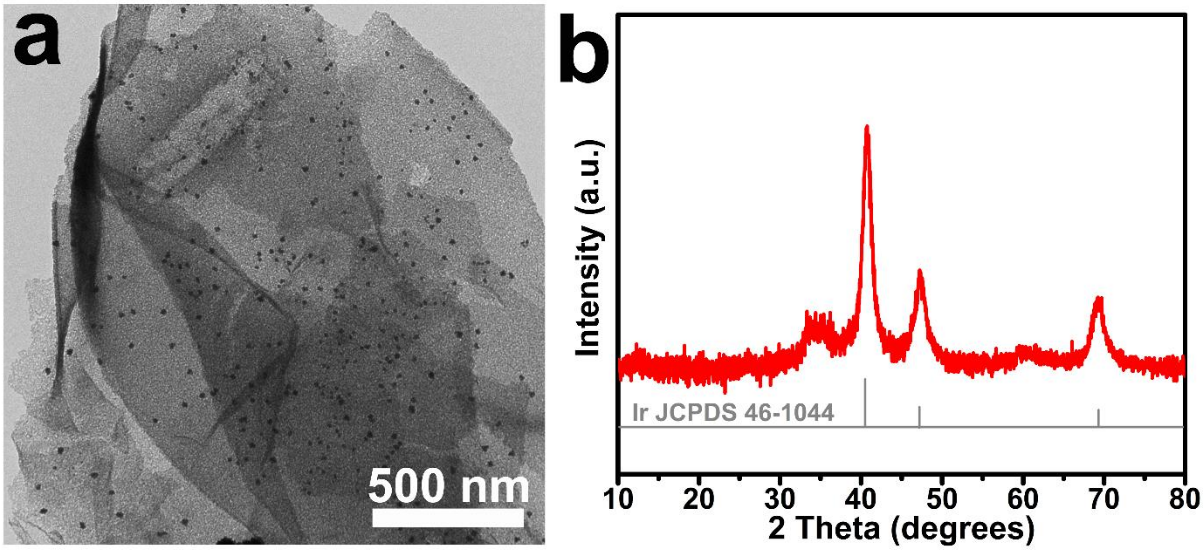

Supplementary Figure 19. Characterizations of crystalline product by annealing at 360 °C. (a) TEM image and (b) XRD pattern of product obtained by directly annealing the mixture of $Ir(acac)_3$ and KNO_3 at 360 °C.

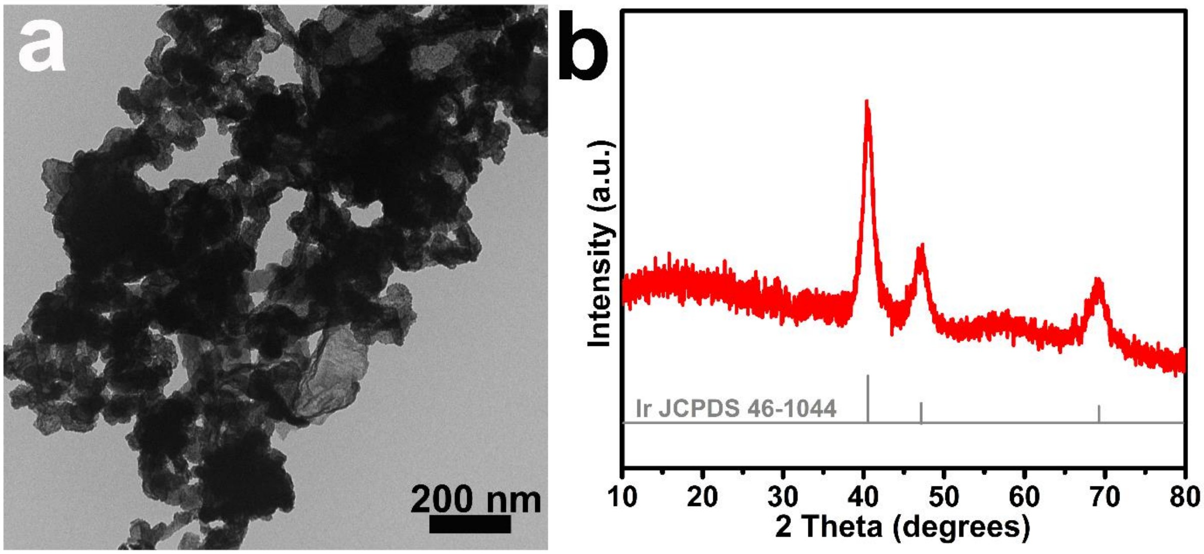

Supplementary Figure 20. Characterizations of crystalline Ir nanoparticles. (a) TEM image and (b) XRD pattern of crystalline Ir nanoparticles.

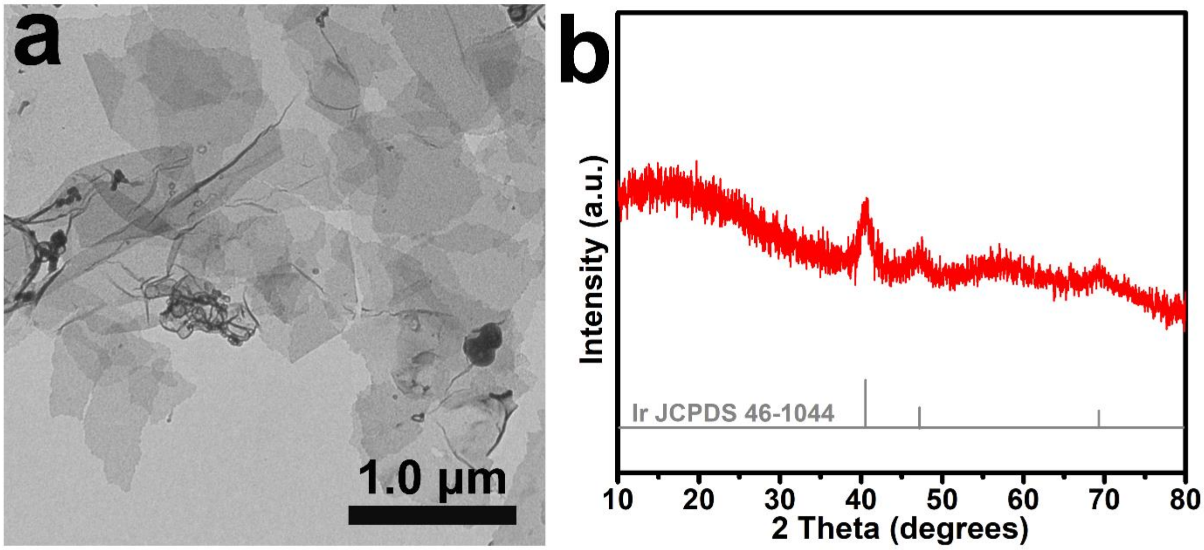

Supplementary Figure 21. Characterizations of crystalline Ir NSs. (a) TEM image and (b) XRD pattern of crystalline Ir NSs obtained by annealing the mixture of $Ir(acac)_3$ and NaCl.

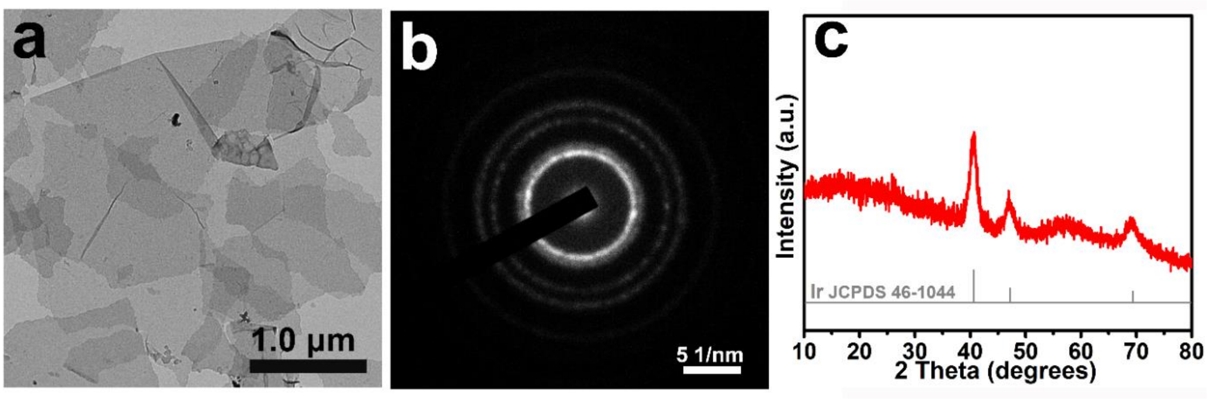

Supplementary Figure 22. Characterizations of crystalline Ir NSs. (a) TEM image, (b) SAED pattern and (c) XRD pattern of crystalline Ir NSs obtained by annealing the mixture of $Ir(acac)_3$ and KBr.

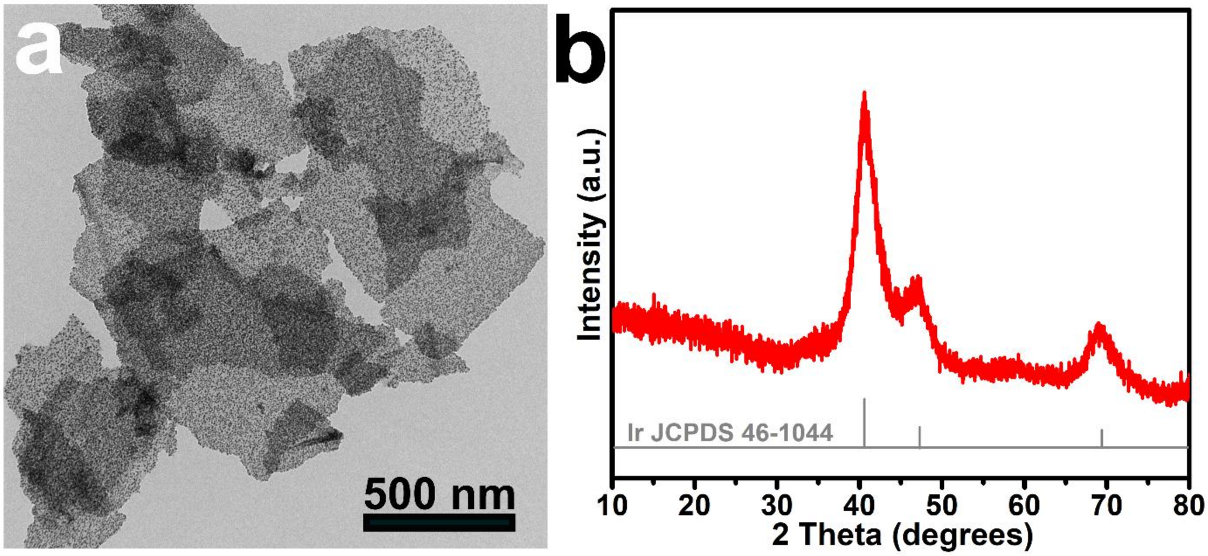

Supplementary Figure 23. Characterizations of carbon supported crystalline Ir nanoparticles. (a) TEM image and (b) XRD pattern of carbon supported crystalline Ir nanoparticles.

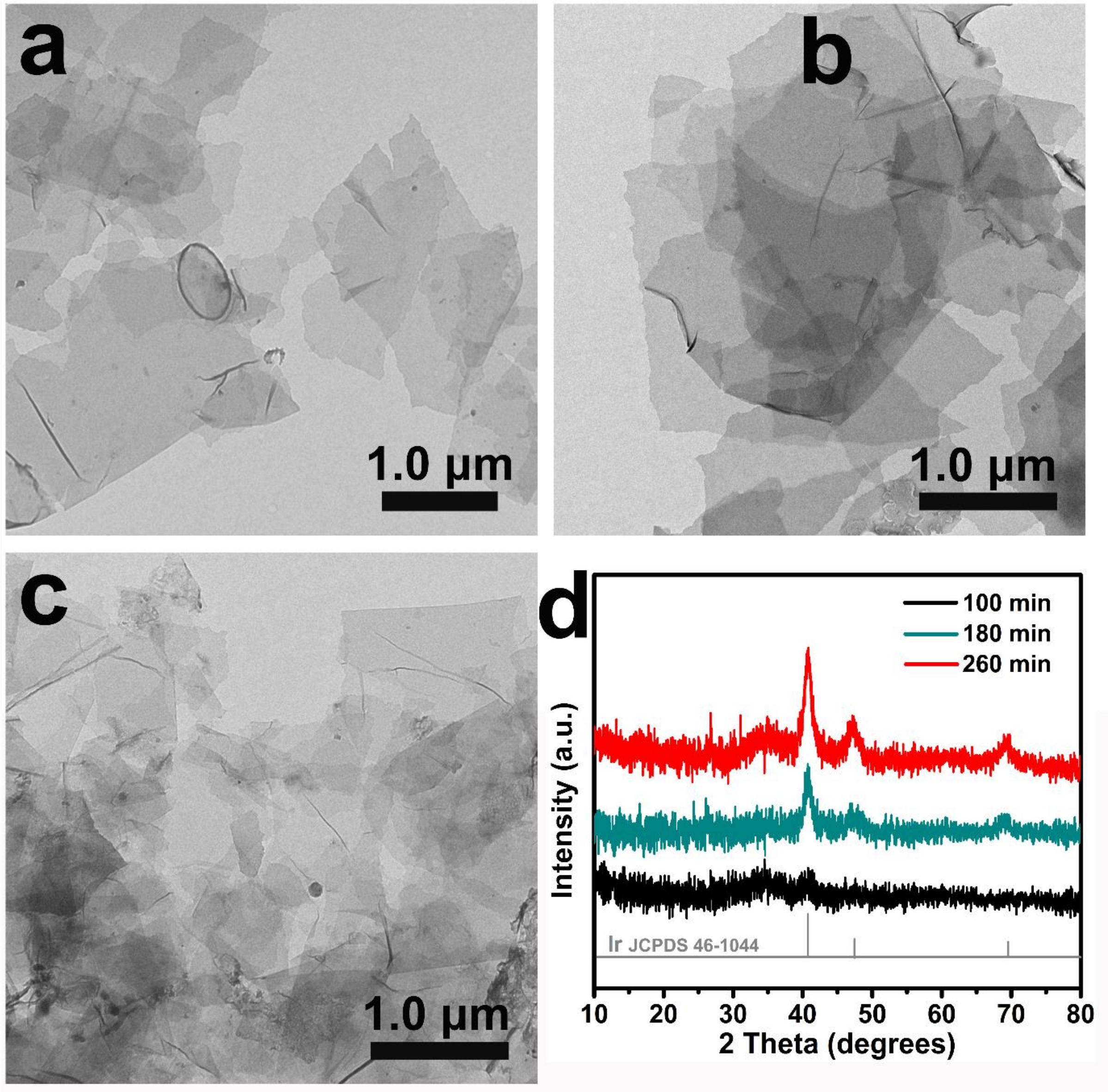

Supplementary Figure 24. Characterizations of product obtained by extending heat time. TEM images of product obtained by annealing for (a) 100 min, (b) 180 min and (c) 260 min. (d) XRD pattern of product obtained.

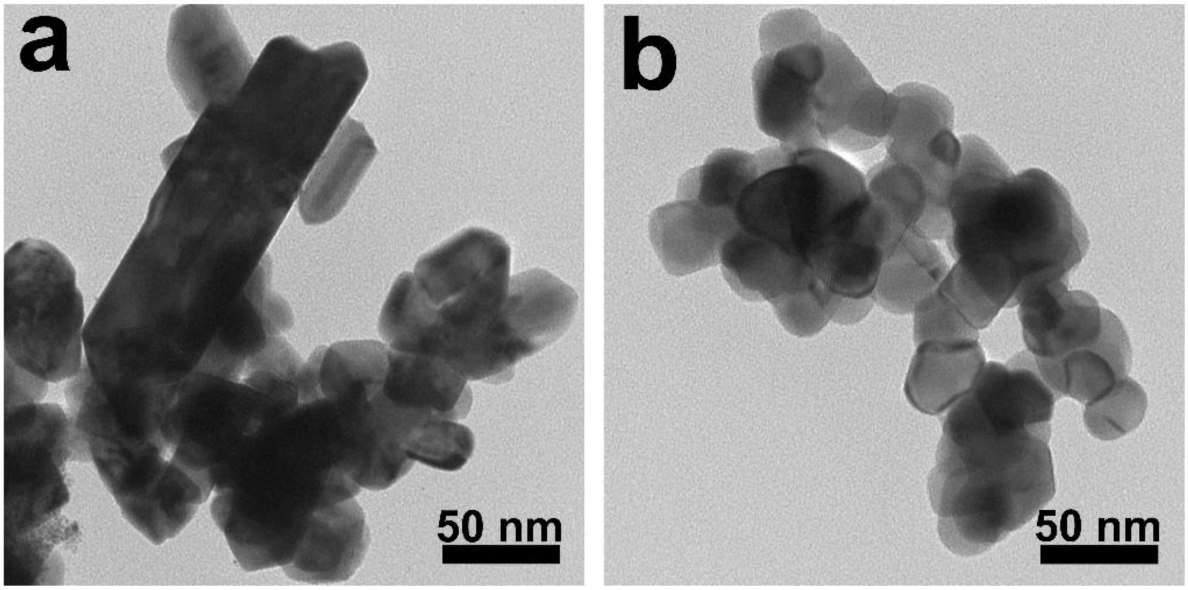

Supplementary Figure 25. Characterizations of commercial catalysts. TEM images of commercial (a) IrO_2 and (b) RuO_2 catalysts.

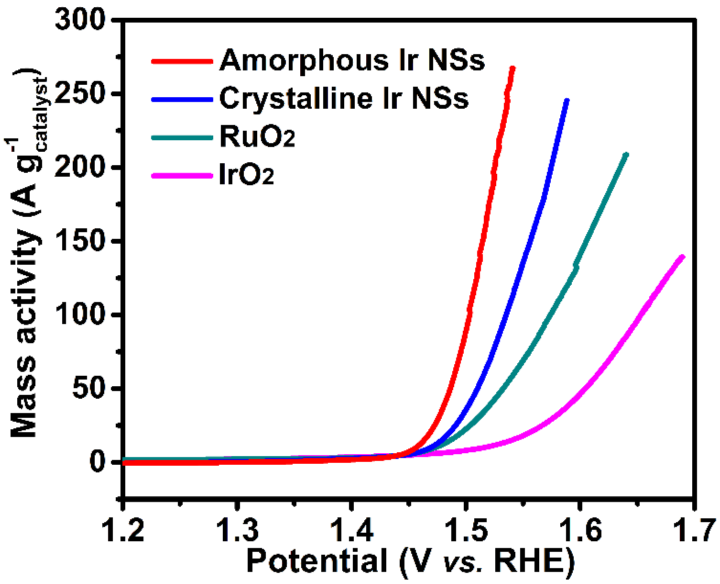

Supplementary Figure 26. Mass activity of amorphous Ir NSs, crystalline Ir NSs, commercial RuO_2 and IrO_2 catalysts at various applied potentials.

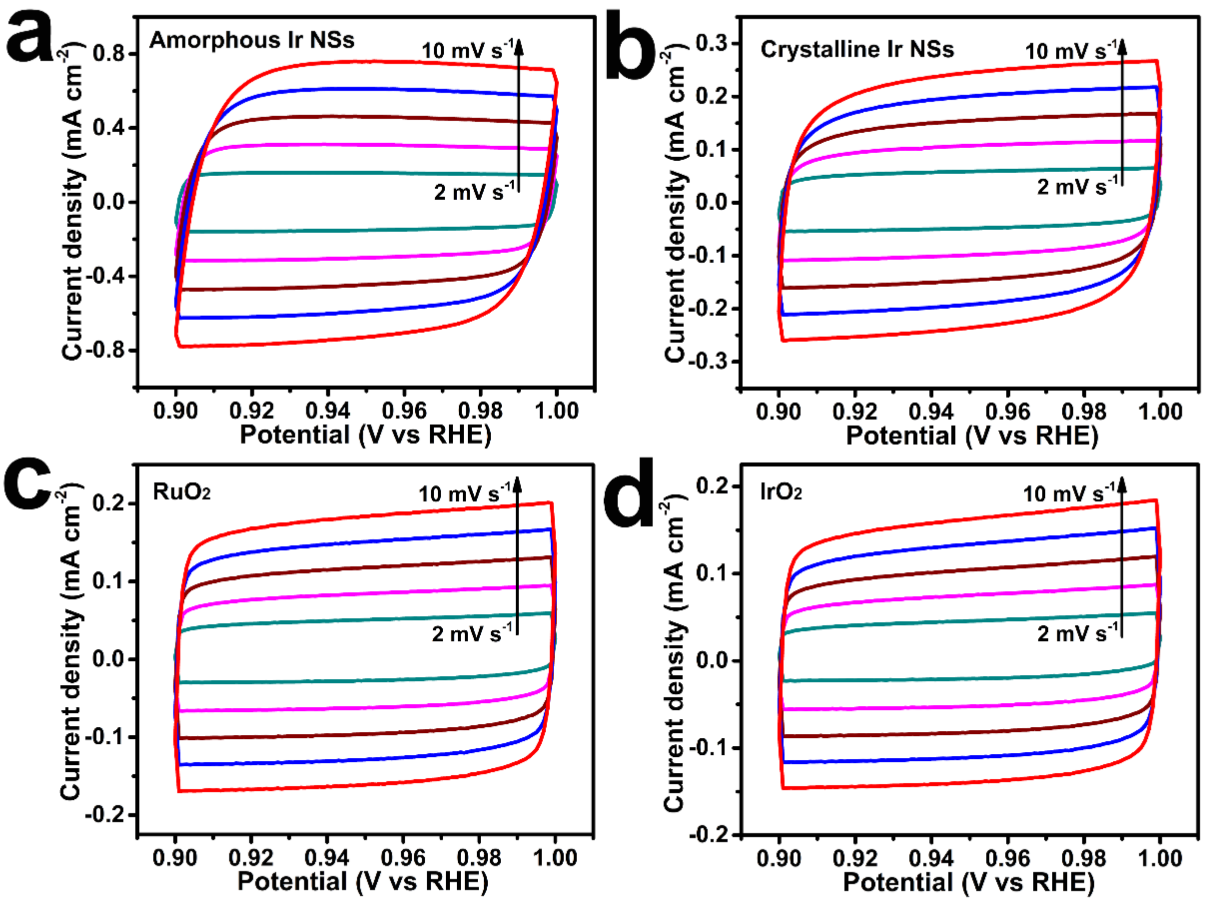

Supplementary Figure 27. CV curves of catalysts. CV curves of (a) amorphous Ir NSs, (b) crystalline Ir NSs, commercial RuO_2 and (d) IrO_2 catalysts in N_2-saturated 0.1 M $HClO_4$ solution, respectively. The scan rate of the CV curves are obtained with a series of scan rate of 2 mV s^{-1} (dark cyan), 4 mV s^{-1} (magenta), 6 mV s^{-1} (wine), 8 mV s^{-1} (blue) and 10 mV s^{-1} (red), respectively.

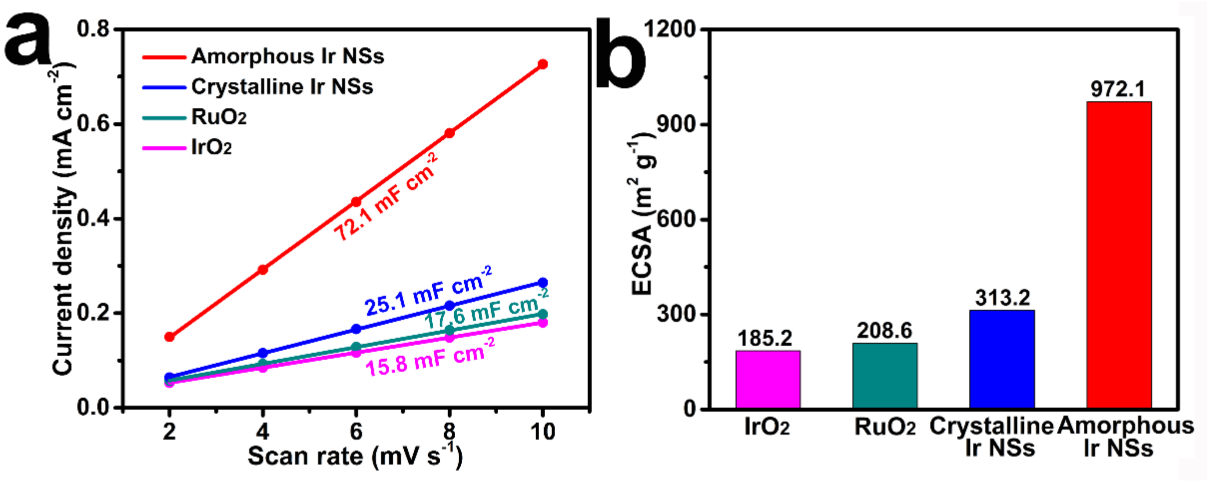

Supplementary Figure 28. ECSA characterization of catalysts. (a) The current density measured at 0.99V vs. RHE plotted as a function of scan rate. (b) The as-calculated ECSA of amorphous Ir NSs, crystalline Ir NSs, commercial RuO_2 and IrO_2 catalysts, respectively.

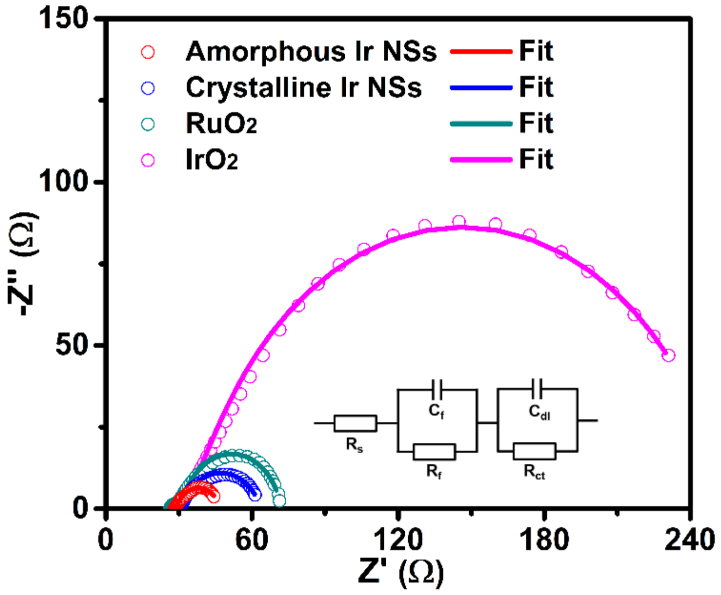

Supplementary Figure 29. Nyquist plots of catalysts at an overpotential of 275 mV. Solid curves are the fitting results by using the equivalent circuit shown in the inset. *Z′* is real impedance and *Z″* is imaginary impedance. The electrolyte resistance (R_s) of amorphous Ir NSs, crystalline Ir NSs, RuO_2 and IrO_2 catalysts is 27.2 Ω, 28.1 Ω, 27.5 Ω and 28.0 Ω, respectively. The transfer resistance (R_{ct}) of amorphous Ir NSs, crystalline Ir NSs, RuO_2 and IrO_2 catalysts is 22.4 Ω, 33.0 Ω, 39.9 Ω and 197.2 Ω, respectively.

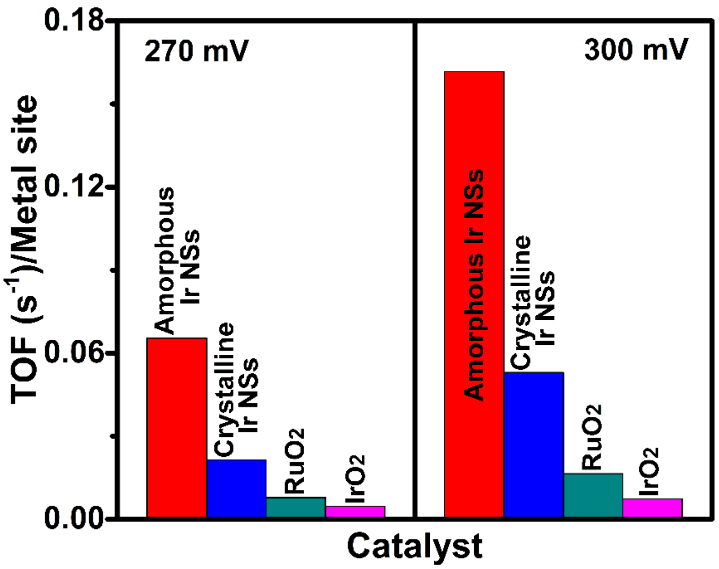

Supplementary Figure 30. TOFs of catalysts at different applied potentials.

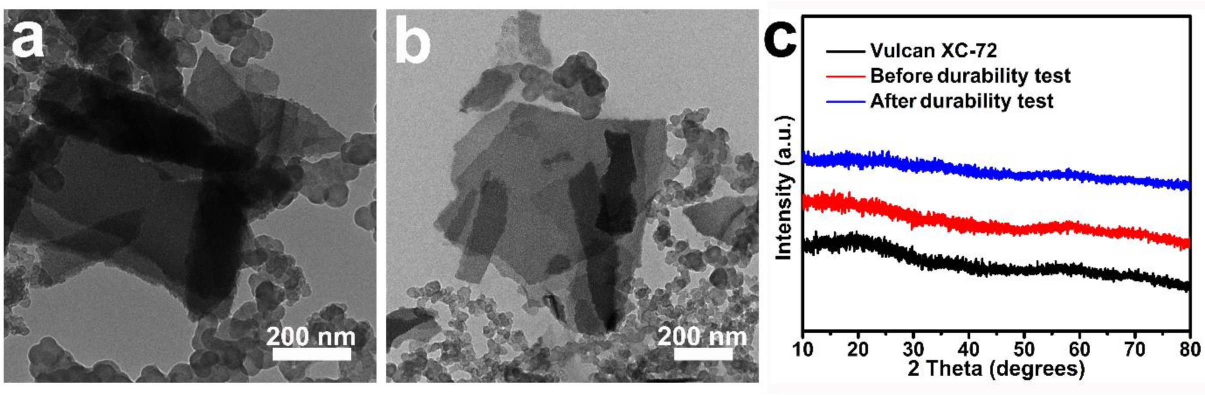

Supplementary Figure 31. Characterizations of amorphous Ir NSs before and after durability test. TEM images of the amorphous Ir NSs (a) before and (b) after durability test, (c) XRD pattern of amorphous Ir NSs before and after durability test, respectively.

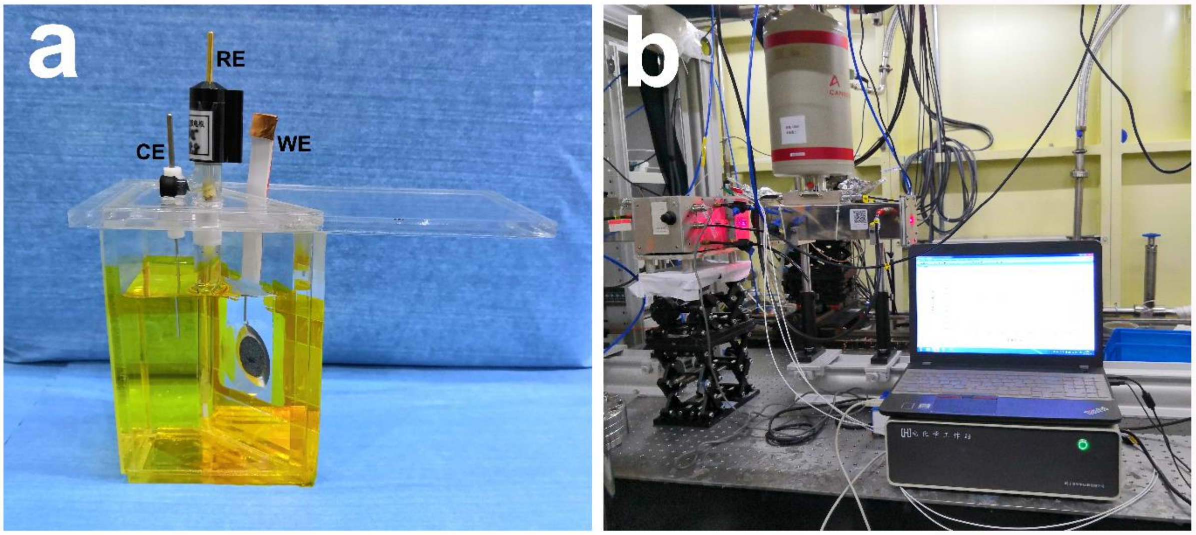

Supplementary Figure 32. Optical images of in-situ XAFS measurement. (a) The electrochemical cell assembly parts. (b) The device and beam path for in-situ XAFS measurement. The experiments were carried out at BL14W1 in SSRF (Ir L_3-edge, Lytle detector was applied).

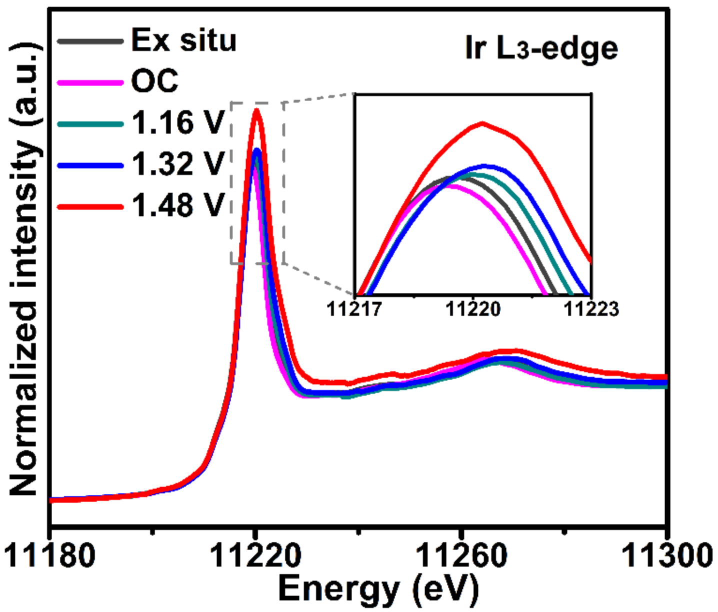

Supplementary Figure 33. In situ XANES spectrum change of the Ir L_3-edge for amorphous Ir NSs under different tested potentials.

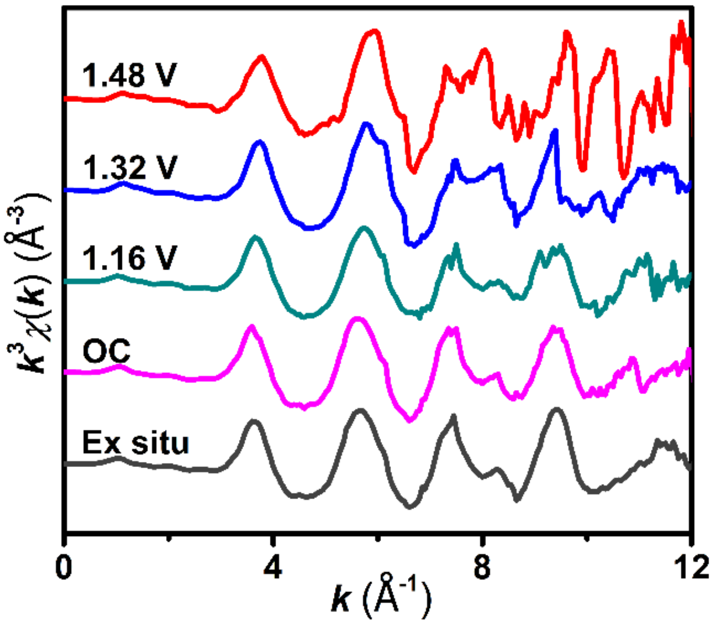

Supplementary Figure 34. In situ EXAFS oscillations change of the Ir L_3-edge for amorphous Ir NSs under different tested potentials.

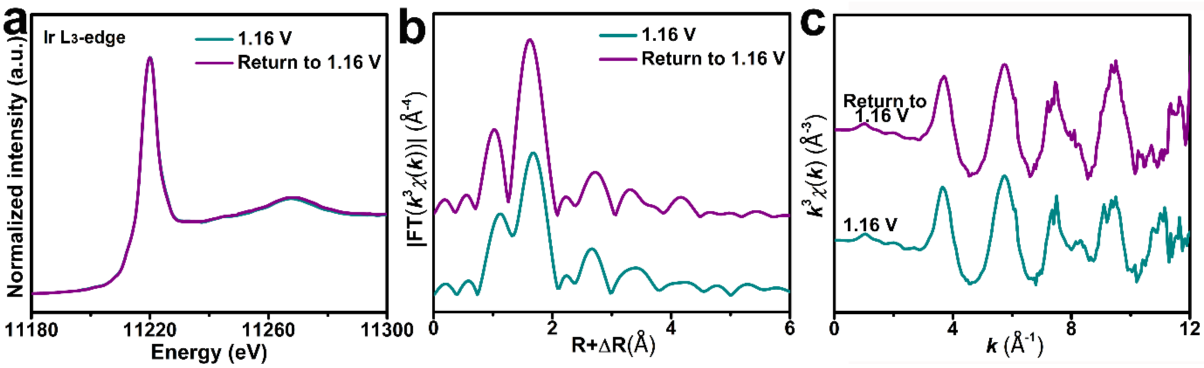

Supplementary Figure 35. XAFS spectra of amorphous Ir NSs after returning to the original potential. a) XANES spectrum, (b) Fourier transforms of k^3-weighted Ir L_3-edge EXAFS experimental data, (c) Ir L_3-edge EXAFS oscillations after returning to the original potential.

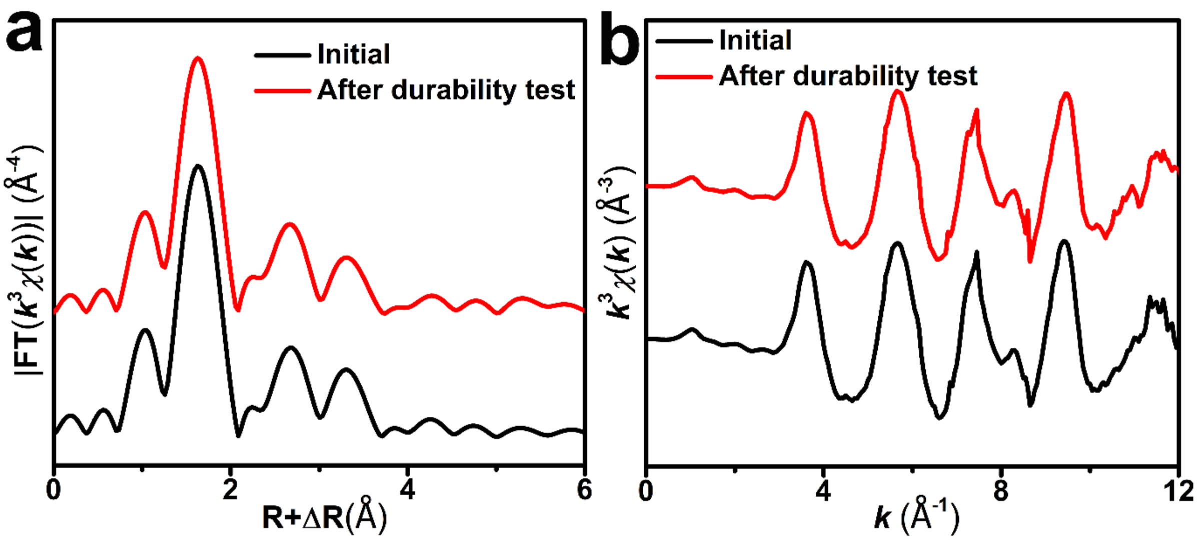

Supplementary Figure 36. XAFS spectra of amorphous Ir NSs after durability test. a) Fourier transforms of k^3-weighted Ir L_3-edge EXAFS experimental data and (b) Ir L_3-edge EXAFS oscillations after durability test.

Supplementary Table 1. Synthetic method of amorphous noble metal NSs.

<table>
<tr><th colspan="2" rowspan="2">Type</th><th colspan="2">Precursor</th><th rowspan="2">Temperature</th><th rowspan="2">time</th><th rowspan="2">gas</th></tr>
<tr><th>Metal precursor</th><th>Inorganic salt</th></tr>
<tr><td rowspan="16">Amorphous nanosheets</td><td>Ir NSs</td><td>$Ir(acac)_3$</td><td>KNO_3</td><td>300 °C</td><td rowspan="16">90 min</td><td rowspan="16">Air</td></tr>
<tr><td>Rh NSs</td><td>$Rh(acac)_3$</td><td rowspan="2">KBr</td><td rowspan="4">290 °C</td></tr>
<tr><td>Ru NSs</td><td>$Ru(acac)_3$</td></tr>
<tr><td>IrRh NSs</td><td>$Ir(acac)_3$, $Rh(acac)_3$</td><td rowspan="5">KNO_3</td></tr>
<tr><td>IrRu NSs</td><td>$Ir(acac)_3$, $Ru(acac)_3$</td></tr>
<tr><td>IrCo NSs</td><td>$Ir(acac)_3$, $Co(acac)_3$</td><td rowspan="4">280 °C</td></tr>
<tr><td>IrFe NSs</td><td>$Ir(acac)_3$, $Fe(acac)_3$</td></tr>
<tr><td>IrNi NSs</td><td>$Ir(acac)_3$, $Ni(acac)_2$</td></tr>
<tr><td>RhRu NSs</td><td>$Rh(acac)_3$, $Ru(acac)_3$</td><td rowspan="7">KBr</td></tr>
<tr><td>RhCo NSs</td><td>$Rh(acac)_3$, $Co(acac)_3$</td><td rowspan="6">270 °C</td></tr>
<tr><td>RhFe NSs</td><td>$Rh(acac)_3$, $Fe(acac)_3$</td></tr>
<tr><td>RhNi NSs</td><td>$Rh(acac)_3$, $Ni(acac)_3$</td></tr>
<tr><td>RuCo NSs</td><td>$Ru(acac)_3$, $Co(acac)_2$</td></tr>
<tr><td>RuFe NSs</td><td>$Ru(acac)_3$, $Fe(acac)_3$</td></tr>
<tr><td>RuNi NSs</td><td>$Ru(acac)_3$, $Ni(acac)_2$</td></tr>
<tr><td>IrRhRu NSs</td><td>$Ir(acac)_3$, $Rh(acac)_3$, $Rh(acac)_3$,</td><td>KNO_3</td><td>280 °C</td></tr>
</table>

Supplementary Table 2. Summary of some recently reported representative OER electrocatalysts in acidic electrolytes.

**Catalysts**	**Electrolyte**	**Catalyst loading amount ($mg\ cm^{-2}$)**	**Substrate**	**Current density ($mA\ cm^{-2}$)**	**Overpotential (mV)**	**Tafel slope ($mV\ dec^{-1}$)**	**Ref.**
Amorphous Ir nanosheets	0.1 M $HClO_4$	0.2	GCE	10	255	40	This work
amorphous Li−IrO_x	0.5 M H_2SO_4	0.05 (Ir)	GCE	10	290	39	1
IrNiCu double-layered nanoframe	0.1 M $HClO_4$	0.02 (Ir)	RDE	10	303	48	2
$IrNi_2$ nanoparticles	0.05 M H_2SO_4	0.0245 (Ir)	GCE	10	356	—	3
amorphous a-IrO_x film	1 M H_2SO_4	0.0001	FTO	10	220	34	4
Co-IrCu nanocages	0.1M $HClO_4$	0.02 (Ir)	GCE	10	293	50	5
$Cu_{1.11}Ir$ nanocages	0.05M H_2SO_4	0.01	GCE	10	286	43.8	6
$Cu_{0.3}Ir_{0.7}O_\delta$	0.1M $HClO_4$	0.2	Ti plate	10	351	63	7
Ir films/Au	0.1M $HClO_4$	—	RDE	5	330	—	8
$Y_2Ru_2O_{7-\delta}$	0.1M $HClO_4$	0.025	RDE	1	270	55	9
$IrCo_{0.65}$ nanodendrites	0.1 M $HClO_4$	0.09	GCE	10	281	59.3	10
Ir-Ni oxide film	0.1 M $HClO_4$	—	RDE	10	307	—	11
$IrNi_x$@IrO_x nanoparticles	0.05M H_2SO_4	0.0102 (Ir)	RDE	1	280	—	12
IrO_2@RuO_2	0.5 M H_2SO_4	0.38	gold slab	10	270	57.8	13
IrO_x-Ir	0.5 M H_2SO_4	0.1333 (Ir)	RDE	10	295	44.7	14
IrO_x/$SrIrO_3$	0.5 M H_2SO_4	—	Copper wires	10	340	—	15
nanoporous IrO_2	0.5 M H_2SO_4	0.379	GCE	10	282	61.8	16
r-IrO_2 nanoparticles	0.1 M $HClO_4$	0.05	GCE	10	345	—	17
macroporous IrO_2	0.5 M H_2SO_4	0.051	RDE	0.5	240	—	18
3D Ir superstructure	0.1 M $HClO_4$	0.0115 (Ir)	GCE	10	270	40.8	19

Note：GCE: Glassy carbon electrode. RDE: Rotating disc electrode

Supplementary references

1 Gao, J. *et al.* Breaking Long-Range Order in Iridium Oxide by Alkali Ion for Efficient Water Oxidation. *J. Am. Chem. Soc.* **141**, 3014-3023 (2019).

2 Park, J. *et al.* Iridium-based multimetallic nanoframe@ nanoframe structure: an efficient and robust electrocatalyst toward oxygen evolution reaction. *ACS Nano* **11**, 5500-5509 (2017).

3 Pi, Y., Shao, Q., Zhu, X. & Huang, X. Dynamic Structure Evolution of Composition Segregated Iridium-Nickel Rhombic Dodecahedra toward Efficient Oxygen Evolution Electrocatalysis. *ACS Nano* **12**, 7371-7379 (2018).

4 Smith, R. D., Sporinova, B., Fagan, R. D., Trudel, S. & Berlinguette, C. P. Facile photochemical preparation of amorphous iridium oxide films for water oxidation catalysis. *Chem. Mater.* **26**, 1654-1659 (2014).

5 Kwon, T. *et al.* Cobalt assisted synthesis of IrCu hollow octahedral nanocages as highly active electrocatalysts toward oxygen evolution reaction. *Adv. Funct. Mater.* **27**, 1604688 (2017).

6 Wang, C. *et al.* Synthesis of Cu–Ir nanocages with enhanced electrocatalytic activity for the oxygen evolution reaction. *J. Mater. Chem. A* **3**, 19669-19673 (2015).

7 Sun, W., Song, Y., Gong, X.-Q., Cao, L.-m. & Yang, J. An efficiently tuned d-orbital occupation of IrO_2 by doping with Cu for enhancing the oxygen evolution reaction activity. *Chem. Sci.* **6**, 4993-4999 (2015).

8 Ahn, S. H. *et al.* Self-terminated electrodeposition of iridium electrocatalysts. *Energy Environ. Sci.* **8**, 3557-3562 (2015).

9 Kim, J. *et al.* High-performance pyrochlore-type yttrium ruthenate electrocatalyst for oxygen evolution reaction in acidic media. *J. Am. Chem. Soc.* **139**, 12076-12083 (2017).

10 Fu, L., Zeng, X., Cheng, G. & Luo, W. IrCo Nanodendrite as an Efficient Bifunctional Electrocatalyst for Overall Water Splitting under Acidic Conditions. *ACS Appl. Mater. Interfaces* **10**, 24993-24998 (2018).

11 Reier, T. *et al.* Molecular insight in structure and activity of highly efficient, low-Ir Ir–Ni oxide catalysts for electrochemical water splitting (OER). *J. Am. Chem. Soc.* **137**, 13031-13040 (2015).

12 Nong, H. N. *et al.* Oxide‐Supported IrNiOx Core‐Shell Particles as Efficient, Cost‐Effective, and Stable Catalysts for Electrochemical Water Splitting. *Angew. Chem. Int. Ed.* **54**, 2975-2979 (2015).

13 Audichon, T. *et al.* IrO_2 coated on RuO_2 as efficient and stable electroactive nanocatalysts for electrochemical water splitting. *J. Phys. Chem. C* **120**, 2562-2573 (2016).

14 Lettenmeier, P. *et al.* Nanosized IrO_x-Ir Catalyst with Relevant Activity for Anodes of Proton Exchange Membrane Electrolysis Produced by a Cost‐Effective Procedure. *Angew. Chem.* **128**, 752-756 (2016).

15 Seitz, L. C. *et al.* A highly active and stable IrO_x/$SrIrO_3$ catalyst for the oxygen evolution reaction. *Science* **353**, 1011-1014 (2016).

16 Li, G. *et al.* Nanoporous IrO_2 catalyst with enhanced activity and durability for water oxidation owing to its micro/mesoporous structure. *Nanoscale* **9**, 9291-9298 (2017).

17 Lee, Y., Suntivich, J., May, K. J., Perry, E. E. & Shao-Horn, Y. Synthesis and activities of rutile IrO_2 and RuO_2 nanoparticles for oxygen evolution in acid and alkaline solutions. *J. Phys. Chem. Lett.* **3**, 399-404 (2012).

18 Hu, W., Wang, Y., Hu, X., Zhou, Y. & Chen, S. Three-dimensional ordered macroporous IrO_2 as electrocatalyst for oxygen evolution reaction in acidic medium. *J. Mater. Chem.* **22**, 6010-6016 (2012).

19 Pi, Y., Zhang, N., Guo, S., Guo, J. & Huang, X. Ultrathin laminar Ir superstructure as highly efficient oxygen evolution electrocatalyst in broad pH range. *Nano Lett.* **16**, 4424-4430 (2016).
